# Impact of intercontinental pollution transport on North American ozone air pollution: an HTAP phase 2 multi-model study

**DOI:** 10.5194/acp-17-5721-2017

**Published:** 2017-05-08

**Authors:** Min Huang, Gregory R. Carmichael, R. Bradley Pierce, Duseong S. Jo, Rokjin J. Park, Johannes Flemming, Louisa K. Emmons, Kevin W. Bowman, Daven K. Henze, Yanko Davila, Kengo Sudo, Jan Eiof Jonson, Marianne Tronstad Lund, Greet Janssens-Maenhout, Frank J. Dentener, Terry J. Keating, Hilke Oetjen, Vivienne H. Payne

**Affiliations:** 1 George Mason University, Fairfax, VA, USA; 2 University of Maryland, College Park, MD, USA; 3 University of Iowa, Iowa City, IA, USA; 4 NOAA National Environmental Satellite, Data, and Information Service, Madison, WI, USA; 5 Seoul National University, Seoul, South Korea; 6 European Centre for Medium-Range Weather Forecasts, Reading, UK; 7 National Center for Atmospheric Research, Boulder, CO, USA; 8 Jet Propulsion Laboratory, California Institute of Technology, Pasadena, CA, USA; 9 University of Colorado Boulder, Boulder, CO, USA; 10 Nagoya University, Furo-cho, Chikusa-ku, Nagoya, Japan; 11 Norwegian Meteorological Institute, Oslo, Norway; 12 Center for International Climate and Environmental Research, Oslo, Norway; 13 European Commission, Joint Research Centre, Ispra, Italy; 14 US Environmental Protection Agency, Washington, DC, USA

## Abstract

The recent update on the US National Ambient Air Quality Standards (NAAQS) of the ground-level ozone (O_3_/ can benefit from a better understanding of its source contributions in different US regions during recent years. In the Hemispheric Transport of Air Pollution experiment phase 1 (HTAP1), various global models were used to determine the O_3_ source–receptor (SR) relationships among three continents in the Northern Hemisphere in 2001. In support of the HTAP phase 2 (HTAP2) experiment that studies more recent years and involves higher-resolution global models and regional models’ participation, we conduct a number of regional-scale Sulfur Transport and dEposition Model (STEM) air quality base and sensitivity simulations over North America during May–June 2010. STEM’s top and lateral chemical boundary conditions were downscaled from three global chemical transport models’ (i.e., GEOS-Chem, RAQMS, and ECMWF C-IFS) base and sensitivity simulations in which the East Asian (EAS) anthropogenic emissions were reduced by 20 %. The mean differences between STEM surface O_3_ sensitivities to the emission changes and its corresponding boundary condition model’s are smaller than those among its boundary condition models, in terms of the regional/period-mean (<10 %) and the spatial distributions. An additional STEM simulation was performed in which the boundary conditions were downscaled from a RAQMS (Realtime Air Quality Modeling System) simulation without EAS anthropogenic emissions. The scalability of O_3_ sensitivities to the size of the emission perturbation is spatially varying, and the full (i.e., based on a 100% emission reduction) source contribution obtained from linearly scaling the North American mean O_3_ sensitivities to a 20% reduction in the EAS anthropogenic emissions may be underestimated by at least 10 %. The three boundary condition models’ mean O_3_ sensitivities to the 20% EAS emission perturbations are ~8% (May–June 2010)/~11% (2010 annual) lower than those estimated by eight global models, and the multi-model ensemble estimates are higher than the HTAP1 reported 2001 conditions. GEOS-Chem sensitivities indicate that the EAS anthropogenic NO_*x*_ emissions matter more than the other EAS O_3_ precursors to the North American O_3_, qualitatively consistent with previous adjoint sensitivity calculations.

In addition to the analyses on large spatial–temporal scales relative to the HTAP1, we also show results on subcontinental and event scales that are more relevant to the US air quality management. The EAS pollution impacts are weaker during observed O_3_ exceedances than on all days in most US regions except over some high-terrain western US rural/remote areas. Satellite O_3_ (TES, JPL–IASI, and AIRS) and carbon monoxide (TES and AIRS) products, along with surface measurements and model calculations, show that during certain episodes stratospheric O_3_ intrusions and the transported EAS pollution influenced O_3_ in the western and the eastern US differently. Free-running (i.e., without chemical data assimilation) global models underpredicted the transported background O_3_ during these episodes, posing difficulties for STEM to accurately simulate the surface O_3_ and its source contribution. Although we effectively improved the modeled O_3_ by incorporating satellite O_3_ (OMI and MLS) and evaluated the quality of the HTAP2 emission inventory with the Royal Netherlands Meteorological Institute–Ozone Monitoring Instrument (KNMI–OMI) nitrogen dioxide, using observations to evaluate and improve O_3_ source attribution still remains to be further explored.

## 1 Introduction

Tropospheric ozone (O_3_), a short-lived trace gas with a lifetime ranging from hours in the boundary layer to weeks in the free troposphere, affects tropospheric chemistry, harms human and ecosystem health, and induces climate change on local, regional, and global scales (Jerrett et al., 2009; Smith et al., 2009; Anenberg et al., 2010; Mauzerall and Wang, 2001; Avnery et al., 2011a, b; Shindell et al., 2009, 2013; Bowman and Henze, 2012; Stevenson et al., 2006, 2013; Monks et al., 2015). It has been recognized that the uneven distributions of tropospheric O_3_ can be attributed to the stratosphere as well as local, regional, and distant emission sources, through complicated processes that occur on synoptic and meso- and micro-scales (Task Force on Hemispheric Transport of Air Pollution (HTAP), 2010; National Research Council, 2009; Maas and Grennfelt, 2016). The mitigation of O_3_’s climate and health impacts would benefit from efforts to control the emissions of its precursors from the various emission sources (United Nations Environment Programme and World Meteorological Organization 2011), such as nitrogen oxides (NO_*x*_), carbon monoxide (CO), methane (CH_4_), and non-methane volatile organic compounds (NMVOCs).

Ground-level O_3_ is one of the six criteria air pollutants regulated by the US Environmental Protection Agency (EPA), and the US National Ambient Air Quality Standards (NAAQS) have recently been lowered to 70 ppbv to better protect Americans’ health and the environment. Issues regarding making accurate estimates of the total O_3_ as well as the background O_3_ level, defined as the concentration that is not affected by recent locally emitted or produced anthropogenic pollution (e.g., McDonald-Buller et al., 2011; Zhang et al., 2011; Fiore et al., 2014; Huang et al., 2015), have been recently discussed as part of the implementation of the new US O_3_ standard (US EPA, 2016a, b). This includes assessing the impacts of various components of the background O_3_, such as stratospheric O_3_, local natural sources such as biogenic, lightning, and wildfire emissions, and the long-range transport (LRT) of pollution. The impact of the trans-Pacific pollution transport on US air quality has been evaluated in numerous studies over the past decades (e.g., Fiore et al., 2009; Reidmiller et al., 2009; Zhang et al., 2008, 2009; Huang et al., 2010, 2013a; Lin et al., 2012a, 2015, 2017; US EPA, 2016a). It has been found that the increasing trends of pollution in the upwind continents, especially the populated East Asia (e.g., Zhang et al., 2014; Susaya et al., 2013; Wang et al., 2012), may partially offset the US air quality improvements in recent decades due to the regional and local emission controls (e.g., Jacob et al., 1999; Verstraeten et al., 2015; Ambrose et al., 2011; Wigder et al., 2013; Cooper et al., 2010; Parrish et al., 2009, 2012; Gratz et al., 2014). A better understanding of the processes that determine the O_3_ pollution levels, as well as an improved capability of attributing the air pollution to nearby or distant sources, is needed to assist with designing and implementing effective local emission control strategies to comply with the tighter air quality standards.

Chemical transport models are often used to reproduce and attribute the observed O_3_ levels, including assessing the impacts of the internationally transported O_3_ on the US air quality. In the HTAP modeling experiment phase 1 (HTAP1), various global models with horizontal resolutions ranging from 1° × 1° to 5° × 5°, only around half of which are finer than 3° × 3°, were used to determine the O_3_ source–receptor (SR) relationships among three continents in the Northern Hemisphere in 2001 (Chapter 4 in HTAP, 2010). The global-model-based SR relationships in HTAP1 determined using the emission perturbation approach (i.e., calculating the changes of O_3_ at the receptor regions in response to a 20% reduction in the emission inputs in a given source region) were reported as either monthly 24 h mean values or policy-relevant metrics such as the maximum daily 8 h average (MDA8) for the US (e.g., Fiore et al., 2009; Reidmiller et al., 2009). Large intermodel diversity was found in the simulated total O_3_ and the intercontinentally transported pollution for the chosen SR pairs in the northern midlatitudes, indicating the challenges with model simulations to accurately represent the key atmospheric processes. Multi-model mean results were the foci in these studies, with the assumption that this approach can reduce the uncertainty from the single model estimates for monthly or seasonal means. “Ensemble” model analyses have been suggested by some US stakeholders as one of the methods for helping with the characterization of the background O_3_ components (US EPA, 2016b). Although the multi-model approach can help identify some of the weaknesses of the individual models and may produce more reliable estimates, it is necessary to well understand the uncertainties inherent in using the same set of anthropogenic emissions in all these model simulations. Satellite observations over the regions with limited in situ measurements such as East Asia can be particularly helpful for quantifying such uncertainties.

The 20% emission perturbation in the HTAP1 modeling experiment was chosen to produce a sizeable (i.e., larger than numerical noise) and realistic impact but small enough in the assumed near-linear atmospheric chemistry regime. The scalability of the modeled O_3_ sensitivities to the size of the emission perturbations has been assessed on continental scale (Wu et al., 2009; Fiore et al., 2009; HTAP, 2010; Wild et al., 2012; Emmons et al., 2012). The receptor O_3_ responses to the source-region emission perturbations are found to be fairly linear within ~50% of the perturbations. However, due to the chemical nonlinearity, the full source contribution obtained by linearly scaling the receptor regional-mean O_3_ sensitivity to the 20% reduction in the source region emissions may be underestimated and the scalability depended on seasons and the perturbed emission species. Huang et al. (2013b) investigated the scalability of the O_3_ sensitivity between the southern California–US intermountain west SR pair for May 2010, in which study the southern California anthropogenic emissions were perturbed by multiple amounts of +50, −50, and −100 %. They reported that the scalability of the O_3_ sensitivities changed with the distance from the source regions. Further analyses on the scalability of these modeled O_3_ sensitivities during recent years especially for the East Asia–NAM(North America) SR pair, as well as their spatial variability, are still needed. Furthermore, results generated using the emission perturbation approach need to be compared with those based on the other methods (e.g., tagged tracers and adjoint sensitivity).

Previous studies have demonstrated the advantages of high-resolution chemical transport modeling for understanding SR relationships (e.g., Lin et al., 2010, for Europe and East Asia; Lin et al., 2012a; Huang et al., 2010, 2013a for Asia and NAM). Using observations (satellite, sondes, and aircraft) along with single model simulations, a few studies have reported that the US O_3_ sensitivities to extra-regional sources is time- and region-dependent (e.g., Lin et al., 2012a, b; Langford et al., 2011; Ott et al., 2016), and therefore the necessity of evaluating the extra-regional source impacts on event scale has been emphasized in these studies as well as in US EPA (2016a, b). The HTAP phase 2 (HTAP2) multi-model experiment, initiated in 2012, is designed to advance the understanding of the impact of intercontinental pollution transport during more recent years (i.e., 2008–2010), involving a number of global and regional models’ participation (Galmarini et al., 2017; Koffi et al., 2016). The regional models are anticipated to help connect the analyses over global and regional scales and enable discussions on small spatial (e.g., subcontinental) and temporal scales (i.e., event based analyses). The use of satellite products for identifying the transport events as well as for quantitative model evaluation is also encouraged in the work plan. The HTAP2 modeling experiment was sequentially conducted in two steps. First, similar to the HTAP1 experiment, a group of global models with different resolutions conducted base and emission perturbation sensitivity simulations to determine the pollutants’SR relationships. All models in their base simulations used the same set of harmonized sector-based global anthropogenic emissions developed specifically for the HTAP2 modeling experiment (Janssens-Maenhout et al., 2015). Most of these global models recorded only key chemical species from their base and sensitivity simulations in varied temporal frequencies. Several global models saved the three-dimensional (3-D) chemical fields of more species with a 3 or 6 h interval, which are suitable for being used as regional models’ chemical boundary conditions. In the second step, regional models conducted base and sensitivity simulations to analyze the pollutants’ SR relationships in greater detail. The regional model simulations used the same set of anthropogenic emissions as the global models within their simulation domains, and the chemical boundary conditions in these regional simulations were downscaled from the base and sensitivity simulations from the selected boundary condition model outputs. For regional simulations over the North America and Europe, boundary conditions were mostly taken from a single model such as the ECMWF C-IFS or GEOS-Chem.

This study aims to address (1) the comparison of the O_3_ sensitivities generated from the HTAP2 and HTAP1 experiments, which could help address how the LRT impacts on NAM changed through time; (2) how the refined modeling experiment design in HTAP2 can help advance our understanding of the LRT impacts on NAM, particularly the involvement of regional models and the inclusion of small spatial–temporal scale analyses during high O_3_ episodes that are more relevant to air quality management; and (3) the usefulness of satellite observations for better understanding the sources of uncertainties in the modeled total O_3_ (e.g., from the emission and regional models’ boundary condition inputs) as well as for reducing the uncertainties in some of these model inputs via chemical data assimilation. We performed a number of regional-scale STEM (Sulfur Transport and dEposition Model) base and sensitivity simulations over NAM during May–June 2010, during which period strong trans-Pacific pollution transports were shown to episodically impact the US (Lin et al., 2012a). Extending the HTAP2 regional simulations’ basic setup, the STEM top and lateral chemical boundary conditions were downscaled from three global models’ (i.e., the Seoul National University (SNU)–GEOS-Chem, RAQMS, and the ECMWF C-IFS) base and sensitivity simulations in which the East Asian anthropogenic emissions were reduced. The STEM surface O_3_ sensitivities over the NAM region based on different boundary condition models were intercompared in terms of the regional averages and the spatial patterns on monthly basis and during a selected event identified by satellite O_3_ and CO products. These were also compared with the sensitivities estimated by their corresponding boundary condition models as well as all HTAP2 participating global models and the results from HTAP1.

## 2 Methods

### 2.1 Anthropogenic emission inputs

Identical anthropogenic emissions were used in all global and regional chemical transport models’ base and sensitivity simulations. This monthly varying harmonized sectoral (i.e., power, industry, transportation, residential, shipping, aircraft, and agriculture) emission inventory was provided on a gridded 0.1° × 0.1° resolution for the years of 2008 and 2010, by compiling the officially reported emissions at the national scale (Janssens-Maenhout et al., 2015; http://edgar.jrc.ec.europa.eu/htap_v2). The temporal profiles for developing the monthly varying emissions differ by region and sector. The amount of emissions of key O_3_ precursors (CO, NO_*x*_, and NMVOCs) from both years are summarized in Table S1 in the Supplement for the four major emissions sectors, over NAM (US + Canada, based on data from the US EPA and the Environmental Canada, which shows lower emissions from the previous years as also discussed in Pouliot et al., 2015), the Model Inter-Comparison Study for Asia (MICS-Asia) regions (South, Southeast, and East Asia, based on country inventory for China and from the Clean Air Policy Support System and the Regional Emission inventory in ASia 2.1, more information also in Li et al., 2017), and for over the world. For all of these species, global total emissions in 2008 and 2010 are similar. The NO_*x*_, NMVOC, and CO emissions decreased from 2008 to 2010 over NAM by 10.7, 9.4, and 15.7 %, respectively. In 2008, NAM NO_*x*_, NMVOC, and CO contributed to 18.0, 11.7, and 11.9% of the global total, respectively, and in 2010 these contributions became 15.8, 10.5, and 10.2 %. For 2010, the transportation sector contributed more than the other sectors to NAM anthropogenic NO_*x*_ and CO emissions; the industrial sector contributed more than the other sectors to NMVOCs emissions. Over East Asian countries, these emissions are ~2–5 times higher than the US emissions, and the NO_*x*_, NMVOC, and CO emissions increased over Asia by 7.3, 7.2, and 1.0 %, with the dominant emission sectors in 2010 of transportation, industry, and residential, respectively. For both years, the emissions over the MICS-Asia regions contribute to over 40% of the global emissions. For these key O_3_ precursors, the East Asian countries contribute to 45% (NMVOCs)–70% (NO_*x*_) of the emissions in the MICS-Asia domain in both years, and the South Asian countries contribute to ~22%(NO_*x*_)–34% (NMVOCs) of the MICS-Asia emissions. The uncertainty of the emission estimates differs by emission sector and species: i.e., the emissions from large-scale combustion sources (e.g., NO_*x*_ and CO from power and industry sectors) are less uncertain than those from small-scale and scattered sources (e.g., CO and NMVOCs from transportation and residential sources). Non-anthropogenic emission inputs used in different models’ simulations may differ, and their impacts on the modeled total O_3_ and the SR relationships will be compared in detail in future studies.

### 2.2 Region definitions for the SR study and the model base and sensitivity simulations

#### 2.2.1 Base and 20% emission perturbation simulations from global and regional models

**Figure 1:**
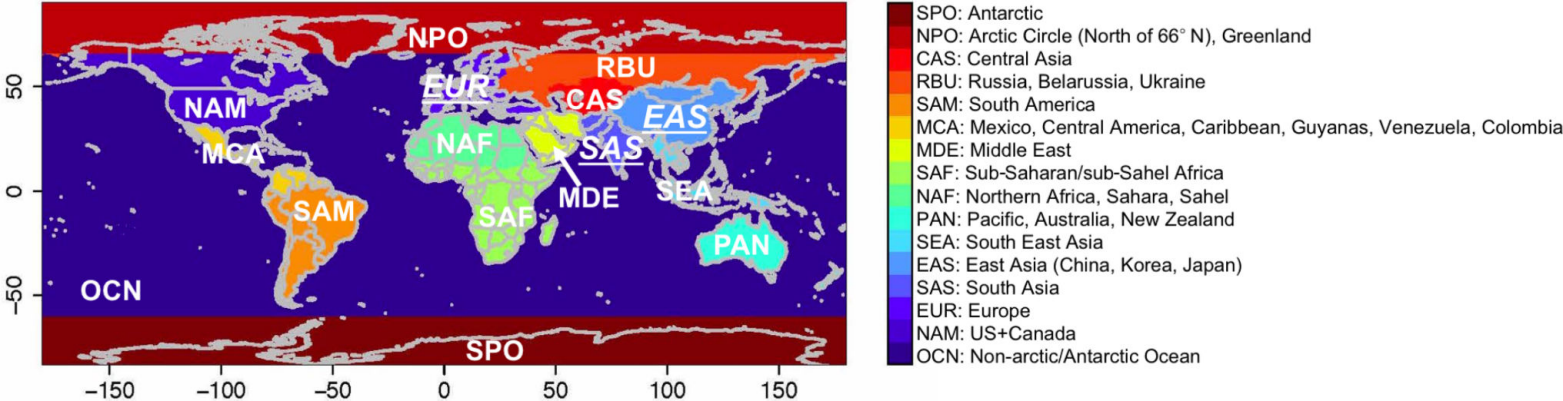
Definitions of the 16 source regions used in the HTAP2 SR relationship study (more details in Koffi et al., 2016). The map is plotted based on data on a 0.1° × 0.1° resolution grid. We focus in this study on the impact of anthropogenic pollution from selected non-North American source regions (i.e., EAS, SAS, and EUR), whose names are underlined and in italic.

The HTAP2 simulations from eight global models, used in this study, are listed in Table 1a, including the relevant references. Horizontal and vertical resolutions of these models range from finer than 1° to coarser than 2.5° and from 20 to 60 layers, respectively. Overall these resolutions are higher than the HTAP1 participating models’. Figure 1 defines the source regions used in the HTAP2 SR relationship study, and we will focus in this study on assessing the East Asia (EAS), South Asia (SAS), Europe (EUR), and non-NAM anthropogenic source (interchangeable in this paper with “(all) foreign”) impacts on the NAM O_3_ levels in 2010. Specifically, each model performed a base simulation and a number of sensitivity simulations in which the original HTAP2 anthropogenic emissions for all species and sectors in a defined source region were perturbed by a certain amount (referring to 20% as in most cases), and these cases are defined in Table 1a–b as [source region]ALL[perturbation (%)], where “ALL” refers to “all species and sectors”, consistent with HTAP1 and HTAP2’s naming convention. The O_3_ differences *R*(O_3_, [source region], [perturbation (%)]) over NAM were then calculated between each model’s base and sensitivity simulations:

(1a) $$R(O_{3},\text{ EAS},\;20\%)=\text{BASE }O_{3}-\text{EASALL}(-20\%)\;O_{3},$$

(1b) $$R(O_{3},\text{ SAS},\;20\%)=\text{BASE }O_{3}-\text{SASALL}(-20\%)\;O_{3},$$

(1c) $$R(O_{3},\text{ EUR},\;20\%)=\text{ BASE }O_{3}-\text{EURALL}(-20\%)\;O_{3},$$

(1d) $$R(O_{3},\text{ non-NAM},\;20\%)=\text{NAMALL}(-20\%)O_{3}-\text{GLOALL}(-20\%)\;O_{3},$$

where “GLO” stands for the “global” source region.

The monthly mean *R*(O_3_, [source region], 20 %) values were averaged over the NAM region for the analysis and compared with the findings in the HTAP1 study (e.g., Fiore et al., 2009). It is worth mentioning that the rectangular source regions defined in HTAP1 were modified in HTAP2 to align with the geopolitical borders. For EAS and SAS, the regions not overlapped by HTAP1 and HTAP2 are mostly in the less populated/polluted regions such as northwestern China, according to the HTAP2 emission maps (http://edgar.jrc.ec.europa.eu/htap_v2/index.php). HTAP2’s EUR domain excludes certain regions in Russia, Belarussia, and Ukraine, the Middle East, and North Africa that are included in HTAP1’s EUR domain. The impact of emissions over these regions on comparing the NAM *R*(O_3_, EUR, 20 %) values in HTAP1 and HTAP2 will be discussed in Sect. 3.2.1.

A unitless Response to Extra-Regional Emission Reductions (RERER) metric (Galmarini et al., 2017), as defined in Eq. (2), was also calculated to measure the importance of local versus non-local sources to NAM’s O_3_ levels:

(2) $$\text{RERER}(O_{3},\text{NAM})=\frac{R(O_{3},\text{non-NAM},20\%)}{R(O_{3},\text{global},20\%)}=\frac{\left(\text{NAMALL}(-20\%)O_{3}-\text{GLOALL}(-20\%)O_{3}\right)}{(\text{BASE}O_{3}-\text{GLOALL}(-20\%)\;O_{3}}.$$

The denominator and numerator terms of RERER represent the impacts of global and non-NAM anthropogenic emissions on NAM O_3_, respectively. The higher the NAM RERER value is, the stronger the impact from non-local sources on NAM is indicated. The RERER value can exceed 1 when emission reductions lead to increasing concentrations (e.g., O_3_ titration by nitrogen monoxide, NO).

**Figure 2:**
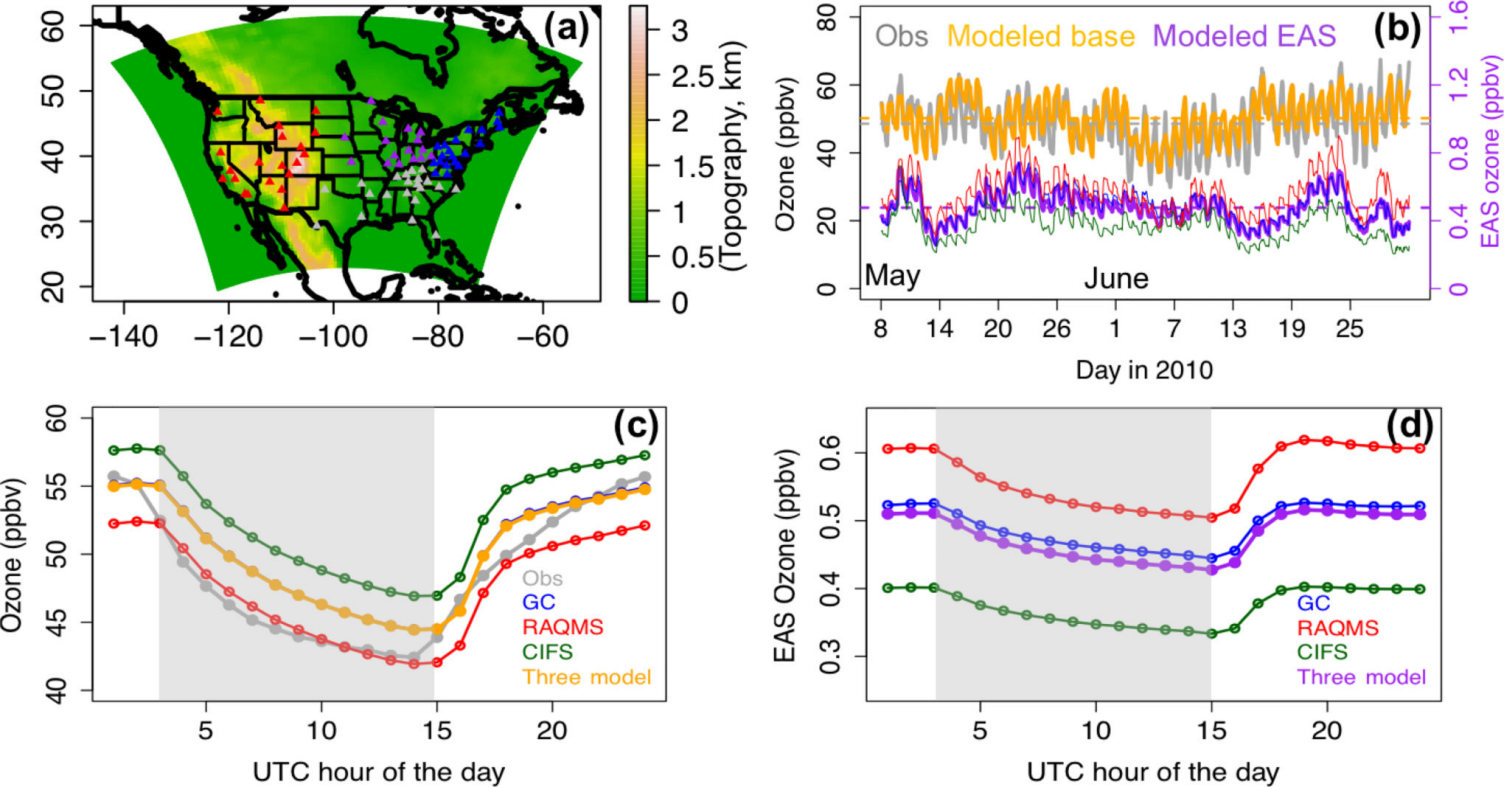
(**a**) The 60 km STEM NAM domain, colored by the model topography. The CASTNET sites to evaluate O_3_ from the STEM base simulations are marked as triangles in different colors that identify the subregions they belong to (red: western US; gray: southern US; purple: the Midwest; blue: northeastern US). (**b**) Evaluation of the STEM-modeled (averaged from the three base simulations using the GEOS-Chem, ECMWF C-IFS, and RAQMS base runs as the chemical boundary conditions) hourly O_3_ at the western US (i.e., EPA regions 8, 9, and 10) CASTNET sites. Observations, modeled base O_3_, and the modeled *R*(O_3_, EAS, 20 %) are in gray, orange, and purple lines, respectively. The horizontal dashed lines indicate the period-mean values. The *R*(O_3_, EAS, 20 %) values from STEM calculations using three different chemical boundary conditions are shown separately in thin lines (blue: GEOS-Chem; red: RAQMS; green: C-IFS). The period-mean diurnal variability of the STEM-modeled (**c**) base and (**d**) *R*(O_3_, EAS, 20 %) at the western US CASTNET sites. The STEM calculations using three different chemical boundary conditions are shown separately as well as averagely. Light gray-shaded areas indicate the local standard nighttime (from 06:00/07:00 p.m. to 07:00/08:00 a.m.).

The STEM (version 2K3) regional simulations were then performed on a 60 km × 60 km horizontal-resolution (a typical coarse regional model resolution) grid over NAM within the domain defined in Fig. 2a during May–June 2010. The meteorological conditions in spring 2010 were compared with the climatology from the NCEP/NCAR reanalysis data for the 1981–2010 period (Kalnay et al., 1996) in Huang et al. (2013b), concluding that this spring represents a period of stronger-than-climatological average spring trans-Pacific transport, based on a stronger meridional gradient of sea level pressure in the North Pacific and higher Pacific/North American (PNA) indexes. This is consistent with the findings by Lin et al. (2014) that the El Niño conditions during the 2009/2010 winter strengthened the trans-Pacific transport of Asian pollution in spring 2010. The mean near-surface air temperatures in the western US in this spring were lower than the climatology, with larger anomalies in the mountain states, which may have led to weaker local O_3_ production and decomposition of the transported peroxyacyl nitrates. In contrast, higher-than-normal temperatures were found in the eastern US that favored anomalously strong local O_3_ production.

STEM has been used to interpret the observations collected by satellites and during aircraft campaigns in the past decade (e.g., Carmichael et al., 2003a, b; Huang et al., 2010, 2013a, b, 2014, 2015). STEM calculates gas-phase chemistry reactions based on the SAPRC 99 gaseous chemical mechanism (Carter, 2000) with thirty photolysis rates calculated online by the Tropospheric Ultraviolet and Visible radiation model (Madronich et al., 2002). Most of the key configurations of the 60 km base simulations are the same as those described in Lapina et al. (2014), i.e., meteorological fields were pre-calculated by the Advanced Research Weather Research and Forecasting Model (WRF-ARW; Skamarock et al., 2008) version 3.3.1 forced by the North American Regional Reanalysis data (Mesinger et al., 2006), using a similar set of the physics configuration to those in Huang et al. (2013a). Biomass burning emissions are from the Fire INventory from NCAR (FINN) version 1.0 (Wiedinmyer et al., 2011). Biogenic emissions were calculated by the Model of Emissions of Gases and Aerosols from Nature (MEGAN) version 2.1 (Guenther et al., 2012), driven by the WRF meteorology. Lightning NO_*x*_ emissions are generated following the method in Allen et al. (2012), with the flash rates determined by the WRF convective precipitation and scaled to the National Lightning Detection Network flash rates. A major difference of the STEM simulations in this study from the Lapina et al. (2014) study is that the anthropogenic emissions were replaced with the monthly mean HTAP2 inventory with no weekday–weekend variability applied, rather than the earlier National Emissions Inventory (NEI) 2005 in which the weekday–weekend variability exists. This change can introduce uncertainty for some US regions where weekday–weekend variability of some O_3_ precursors’ emissions was notable during the studied period (e.g., weekend NO_*x*_ emissions in southern California during CalNex 2010 were 0.6–0.7 of the weekday emissions as reported by Kim et al., 2016, and Brioude et al., 2013), but this was done to ensure consistency with the HTAP2 global model simulations, which also did not use daily variable emissions for any regions in the world. The VOC speciation for the SAPRC 99 chemical mechanism in the NEI 2005 (ftp://aftp.fsl.noaa.gov/divisions/taq/emissions_data_2005) were applied to break down the total NMVOC emissions provided in the HTAP2 inventory. The VOC speciation based on the year of 2005 can be unrealistic for 2005 and 2010, as studies have reported variable temporal changes of different VOC species in some US cities (e.g., Warneke et al., 2012). The time-varying lateral and top boundary conditions in the STEM base simulations were downscaled from three global models’ (i.e., 3-hourly SNU–GEOS-Chem, 3-hourly ECMWF C-IFS, and 6-hourly RAQMS) base simulations. In support of the SR relationship study to quantify the East Asia anthropogenic impacts on NAM, three STEM sensitivity simulations were also conducted in which the STEM boundary conditions were downscaled from the EASALL(−20 %) sensitivity simulations by these three global models (Table 1b). All STEM-simulated 3-D chemical fields were saved hourly for the convenience of calculating the US primary O_3_ standard metric MDA8 as well as the quantitative comparisons against the satellite Level 2 (L2) O_3_ products. The STEM base case surface O_3_ performance and its O_3_ sensitivities were also compared with those of its boundary condition models as well as the multi-global model means. The latitude/longitude ranges (20–50° N/130–65° W) of NAM for the global and regional-model-based sensitivity calculations were selected to mainly account for the coverage of the STEM domain, which are slightly different from the definition of North America in HTAP1.

Note that non-anthropogenic emission inputs used in STEM and its boundary condition models differed, as summarized in Table 1c. Figure S1 in the Supplement shows detailed comparisons between STEM and GEOS-Chem’s non-anthropogenic (i.e., soil, lightning, and biomass burning) NO_*x*_ emission inputs, and their impacts on the modeled NAM background O_3_ were included in Lapina et al. (2014). Such quantitative comparisons will also be carried out between STEM and its other boundary condition models in future studies.

#### 2.2.2 Additional base and sensitivity simulations from selected models

In addition to the base and 20% EAS all-category emission perturbation simulations, the global RAQMS model conducted a sensitivity simulation in which the East Asian anthropogenic emissions were zeroed out, which was also used as STEM’s boundary conditions (Table 1b).We calculate the “*S*_O_3__” metric (Eq. 3) using the O_3_ sensitivities in STEM and RAQMS at the receptor regions in response to both 20 and 100% of emission reductions to explore the relationships between the O_3_ sensitivity and the size of the emission perturbation. A closer-to-one *S*_O_3__ value indicates higher scalability of the sensitivity based on the 20% emission perturbation method for obtaining the full “contribution” of the East Asian anthropogenic emissions on the NAM O_3_.

(3) $$S_{O_{3}}=R(O_{3},\;\text{EAS},\;100\%)/R(O_{3},\;\text{EAS},\;20\%)/5,$$

where *R*(O_3_, EAS, 100 %) = BASE O_3_ − EASALL (−100 %) O_3_.

The RAQMS model also provided a base simulation that assimilated satellite O_3_ products from the Ozone Monitoring Instrument (OMI; Levelt et al., 2006) and Microwave Limb Sounder (MLS; Livesey et al., 2008; Pierce et al., 2007), which was used to help better understand the regional model base run error sources as well as for demonstrating the use of satellite observations to help improve the representation of the trans-boundary pollution.

We also used a number of sensitivity simulations produced by the GEOS-Chem adjoint model v35f in which the emissions from selected anthropogenic emission sectors (power and industry, transportation, residential) or individual O_3_ precursor chemical species (NO_*x*_, VOC, CO) over East Asia were reduced by 20 %. Additional simulations for the 2008–2009 periods by the SNU–GEOS-Chem were also utilized to quantify the East Asia and non-NAM anthropogenic source impacts in comparison to the 2010 conditions that we mainly focus on in this study.

### 2.3 In situ and satellite observations

#### 2.3.1 In situ observations

Over the NAM receptor, the hourly O_3_ observations at the Clean Air Status and Trends Network (CASTNET, http://epa.gov/castnet/javaweb/index.html) sites were used to evaluate the global and regional models’ base simulations in four subregions: western US (i.e., the EPA regions 8, 9, and 10); southern US (i.e., the EPA regions 4 and 6), the Midwest (i.e., the EPA regions 5 and 7), and the northeast (i.e., the EPA regions 1–3). The numbers of sites used in global and regional models’ evaluation in each US subregion are summarized in Tables 2 and 3. The locations of these sites and the subregions they belong to are indicated in Fig. 2a, overlaid on a model-based terrain height map. A majority of the CAST-NET sites in the western US are located at high-elevation (>1 km) remote or rural regions, which are more susceptible to the trans-boundary pollution (e.g., Jaffe, 2011). Most of the sites in the other three subregions are located in low elevation regions, mainly affected by local and regional pollution. The model-based terrain heights represent the reality on subregional scale fairly well – the differences between the actual and model-based subregional-mean terrain heights at the CASTNET sites are smaller than 0.1 km (Table 3).

During May–June 2010, intense ozonesonde measurements were made at multiple California locations (Cooper et al., 2011), in support of the NOAA California Research at the Nexus of Air Quality and Climate Change (CalNex) field experiment (Ryerson et al., 2013). They have been used to evaluate the simulated O_3_ vertical profiles by the HTAP2 participating models. The detailed evaluation results have been shown by Cooper (2016) and will be covered by subsequent publications.

**Figure 3:**
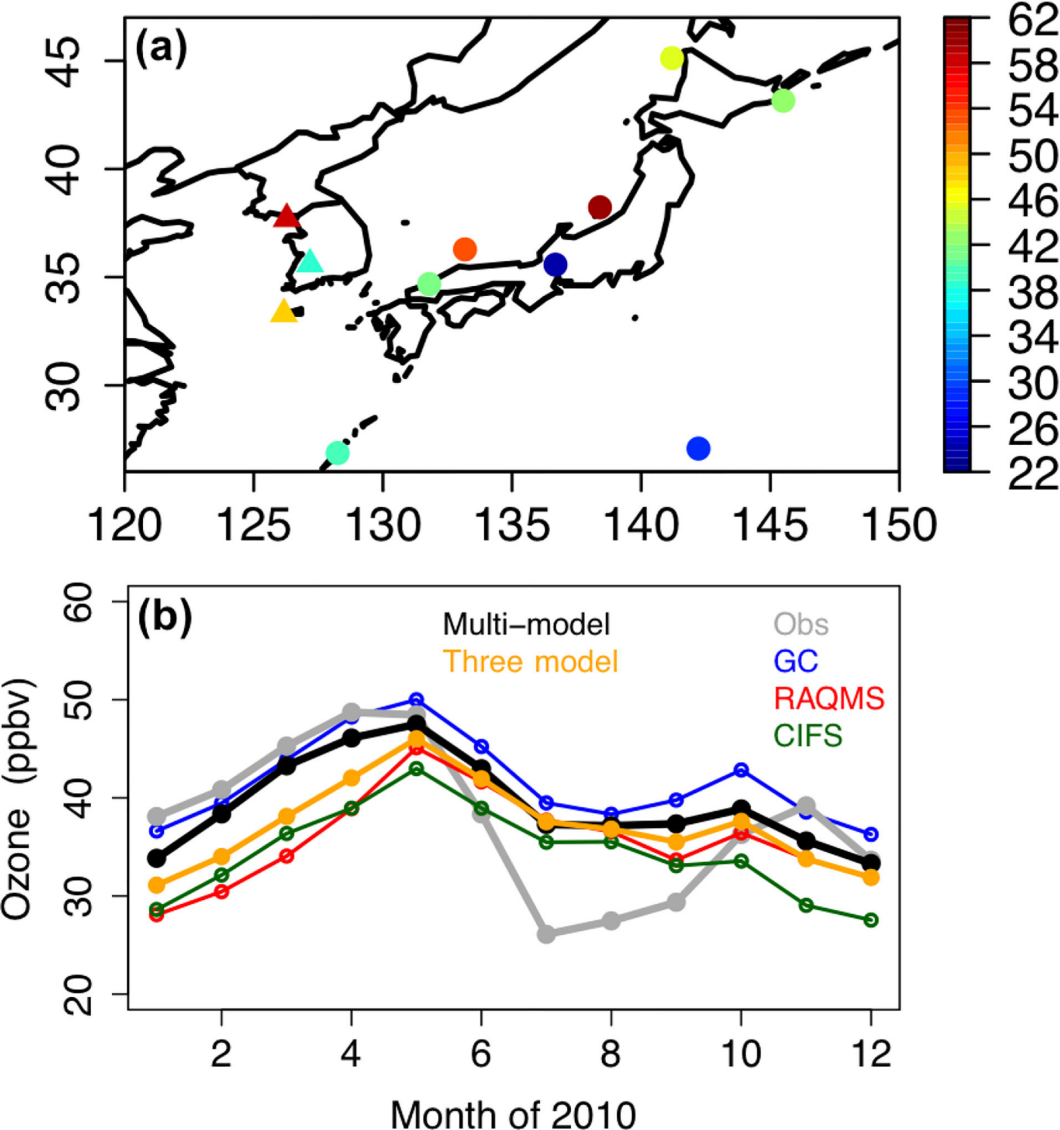
(**a**) May–June 2010 period-mean surface O_3_ observations in ppbv at eight Japanese (filled circles) and three South Korean (filled triangles) EANET sites. (**b**) Observed and modeled monthly mean surface O_3_ in 2010 at all eleven EANET sites. The “Multimodel” and “Three model” in the legend indicate the mean values of all eight global models and only of the three boundary condition models, respectively.

Over HTAP2’s EAS source region, the global models’ O_3_ performance was evaluated against the monthly mean surface in situ O_3_ measurements at 11 sites within the Acid Deposition Monitoring Network in East Asia (EANET, http://www.eanet.asia) that had data throughout the year of 2010. These include eight Japanese and three South Korean sites (Fig. 3a), all of which are located at low elevation regions (2–150 m). The reported monthly mean observations at these sites were based on weekly or daily sampled data, varying among sites.

#### 2.3.2 Satellite products

In two case studies of high O_3_ episodes, L2 and L3 O_3_ and CO retrievals from several satellite instruments were used to assess the impacts of trans-Pacific pollution transport and stratospheric O_3_ intrusions on NAM O_3_ levels in early May. These include (1) the early afternoon O_3_ and CO profiles version 5 from the Tropospheric Emission Spectrometer (TES; Beer et al., 2001; Beer, 2006) on the Aura satellite; (2) the mid-morning O_3_ profiles from the METOP–Infrared Atmospheric Sounding Interferometer (IASI), which were retrieved using the Jet Propulsion Laboratory (JPL) TES optimal estimation retrieval algorithm (Bowman et al., 2006) for selected areas including the western US (Oetjen et al., 2014, 2016); and (3) the early afternoon L3 O_3_ and CO maps (version 6, 1° × 1°) from the Aqua Atmospheric Infrared Sounder (AIRS) instrument. The TES tropospheric O_3_ retrieval is often sensitive to the mid- to lower free troposphere, and O_3_ at these altitudes in the eastern Pacific is known to possibly impact the downwind US surface air quality at later times (Huang et al., 2010; Parrish et al., 2010). TES O_3_ is generally positively biased by <15% relative to high accuracy/precision reference datasets (e.g., Verstraeten et al., 2013). Although IASI is in general less sensitive than TES due to its coarse spectral resolution, the 681–316 hPa partial column-averaged O_3_ mixing ratios in the JPL product agree well with TES O_3_ for the 2008–2011 period with a −3.9 ppbv offset (Oetjen et al., 2016). Note that IASI O_3_ data are processed operationally in Europe using a different algorithm. For this work we used O_3_ profiles from TES and IASI processed using a consistent algorithm at JPL, although the latter set of data represents only a small subset of the full set of the IASI radiance measurements. The IASI and TES L2 O_3_ profiles (screened by the retrieval quality and the C-curve flags) were used to evaluate the STEM O_3_ vertical distributions in the different base simulations, and the satellite observation operators were applied in these comparisons. Taking TES as an example, its observation operator ***h**_z_* for O_3_ is written in Eq. (4):

(4) $$h_{z}=z_{c}+A_{\text{TES}}(\text{ln}(F_{\text{TES}}(c))-z_{c}),$$

where ***z**_c_* is the natural log form of the TES constraint vector (a priori) in volume mixing ratio. **A**_TES_ is the averaging kernel matrix reflecting the sensitivity of retrieval to changes in the true state (Rodgers, 2000). **F**_TES_ projects the modeled O_3_ concentration fields ***c*** to the TES grid using spatial and temporal interpolation. The exponent of ***h**_z_* is then used to compute the mismatches between the model and TES O_3_ retrievals as the model evaluation. A small mismatch between model with the satellite observation operators and the satellite retrievals may indicate either good model performance or may be the low sensitivity of the retrievals to the true O_3_ profile. AIRS O_3_ is sensitive to the altitudes near the tropopause, with positive biases over the ozonesondes in the upper troposphere (e.g., Bian et al., 2007); AIRS CO is most sensitive to 300–600 hPa (Warner et al., 2007) and is frequently used together with the AIRS O_3_ to distinguish the stratospheric O_3_ intrusions from long-range transported anthropogenic or biomass burning pollution. We use the L3 AIRS products in this study to get a broad overview of the areas that are strongly impacted by the stratospheric O_3_ intrusions or/and LRT of pollution.

The bottom-up NO_*x*_ emissions from the HTAP2 inventory were assessed on a monthly base by comparing the GEOS-Chem nitrogen dioxide (NO_2_) columns with the de-striped KNMI (Royal Netherlands Meteorological Institute) OMI column NO_2_ product version 2.0 (Boersma et al., 2011a, b). For this model evaluation against the OMI L2 products, the NO_2_ fields calculated by the GEOS-Chem adjoint model were saved daily at 13:30 local solar time, roughly coinciding with the Aura and Aqua overpassing times. Other parameters used in the model column calculations came from the GEOS-5/GEOS-Chem monthly mean conditions. The OMI data that passed the tropospheric quality flag at 13:00–14:00 local time were selected based on the following screening criteria: surface albedo <0.3, cloud fraction <0.2, solar zenith angle <75°, and viewing zenith angle <45°. The averaging kernels (Eskes and Boersma, 2003) and air mass factors in the KNMI product were used to calculate the modeled tropospheric NO_2_ vertical columns comparable to the OMI. Details of the method to compare the model-based NO_2_ columns with the KNMI–OMI can be found in Huang et al. (2014).

## 3 Results and discussions

### 3.1 Evaluation of the HTAP2 bottom-up NO_*x*_ emissions and the model base simulations

#### 3.1.1 Evaluation of the bottom-up NO_*x*_ emissions

**Figure 4:**
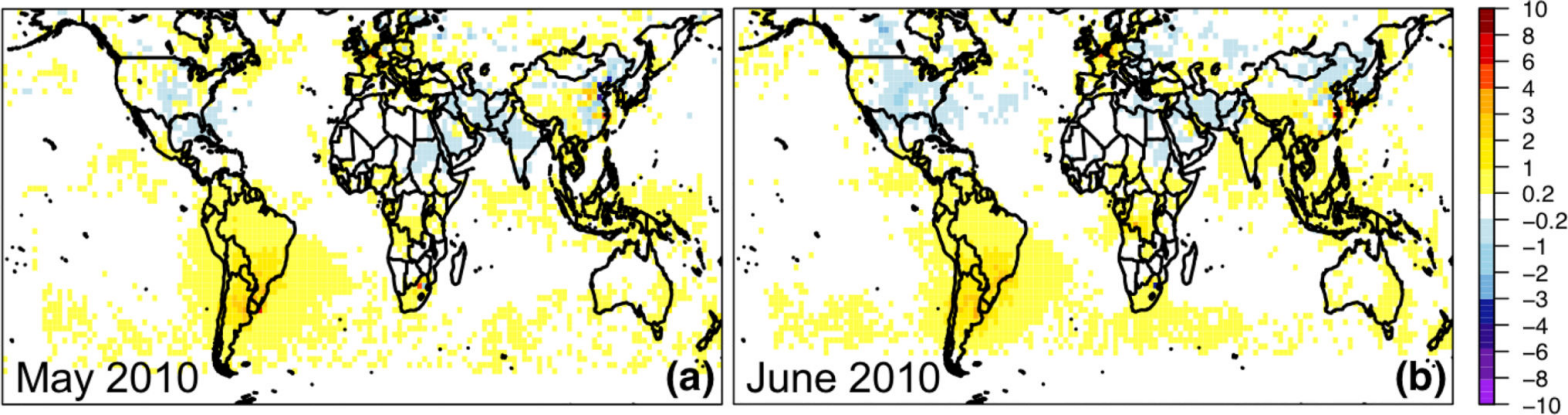
Evaluation of the GEOS-Chem adjoint base NO_2_ product (recorded at near the satellite overpassing time) with the OMI NO_2_ columns. The differences between OMI and GEOS-Chem (OMI-modeled) tropospheric NO_2_ columns (×10^15^ molec. cm^−2^) are shown for (**a**) May and (**b**) June 2010. Details of the comparison are included in Sect. 2.3.2.

The comparison of the GEOS-Chem adjoint NO_2_ columns with the OMI product was used to help assess the bottom-up HTAP2 NO_*x*_ emissions. Figure 4 shows that NO_2_ columns from GEOS-Chem’s base simulations over the US are overall overestimated. While grid-scale differences in NO_2_ columns may not be directly indicative of emissions biases (Qu et al., 2017), these discrepancies are possibly due to a positive bias in the bottom-up emissions, mainly from the anthropogenic sources, which have also been pointed out by Anderson et al. (2014) and Travis et al. (2016). A larger OMI-model disagreement was found over the central and eastern US in June 2010 than in May, likely also due to the uncertainty in GEOS-Chem’s soil or lightning NO_*x*_ emissions, which appear to be high over these regions (Fig. S1). The NO_2_ columns in the GEOS-Chem base simulation were overestimated in many northern China rural areas and underpredicted in a few urban areas in East Asia as well as a broad area in southwestern China. The mismatches between model and OMI NO_2_ fell within the ranges of the comparison between the GOME2 NO_2_ column product and six models’ simulations over China in summer 2008 (Quennehen et al., 2016). Additionally, the use of monthly mean anthropogenic emissions as well as the overall rough treatment of emission height and temporal profiles can be sources of uncertainty. These global model evaluation results suggest that the EAS–NAM SR relationships analyzed using this inventory may overall overestimate the NAM local contribution and underestimate the EAS contribution. Under different chemical regimes, this statement would also rely on the quality of other O_3_ precursors’ emissions in the HTAP2 inventory, and they may be associated with variable uncertainties depending on the species or emission sector as introduced in Sect. 2.1. Therefore, careful assessment of other key O_3_ precursors’ emissions in the inventory is needed in the future work. It is important to note that uncertainty in satellite retrievals can prevent us from producing an accurate assessment on emissions (e.g., van Noije et al., 2006), and this comparison does not account for the biases in the used OMI data and would be further validated by using other OMI NO_2_ products as well as the bias-corrected (if applicable) in situ NO_2_ measurements. We also recommend for more global models to save their calculations more frequently, at least near the satellite overpassing times, for a more comprehensive assessment of the emission inventory and a better understanding of the model biases.

#### 3.1.2 Evaluation of the global model O_3_ performance in NAM and EAS

The monthly mean surface O_3_ from multiple global models’ free runs was evaluated with the CASTNET observations at the stations with 95% of the hourly O_3_ observation completeness for the 1 May–30 June 2010 period. The mean biases and RMSEs for these 2 months were summarized in Table 2a by US subregions. The three boundary condition model as well as the eight-model ensembles overall underpredicted O_3_ in the western US (by ~3–6 ppbv), similar to the HTAP1 model performance over these regions for May–June 2001 presented in Fiore et al. (2009). This can be due to the underestimated trans-boundary pollution (as indicated by the evaluation of modeled O_3_ profiles with ozonesondes and satellite O_3_ products). In addition, the coarser model resolutions are less capable of resolving the local features that influence the pollutants’ import processes, chemical transformation, and regional processes such as the cross-state pollution transport over complex terrains. The global RAQMS base simulation with satellite assimilation improved the free tropospheric O_3_ structure as its comparisons with the ozonesondes shows, which also enhanced the simulated monthly mean surface O_3_ by up to > 10 ppbv in the western US and some coastal areas in the southeastern US (Fig. S2, left). The global models overall significantly overestimated O_3_ in the other three subregions (by 8–12 ppbv), close to HTAP1 model performance for May–June 2001 over the similar areas (Fiore et al., 2009) and in the Lapina et al. (2014) study for 2010, in large part due to the uncertainties in the bottom-up emissions as discussed in Sect. 3.1.1. Satellite assimilation led to 2–6 ppbv higher RAQMS surface O_3_ in the central, southern, and eastern US than in its free simulation, which is associated with higher positive biases.

The surface O_3_ performance by individual global models varies significantly, for example, with the RMSEs at all CASTNET sites ranging from ~9 to > 15 ppbv (Table 2b). As reported in the literature (e.g., Geddes et al., 2016; Travis et al., 2016), the representation of land use/land cover, boundary layer mixing, and chemistry can be sources of uncertainty for a certain global model (i.e., GEOS-Chem), but how serious these issues were in the other models needs to be investigated further. Some other possible reasons include the variation of these models’ non-anthropogenic emission inputs and chemical mechanisms (Table 1c). Future work should emphasize on evaluating and comparing all models on process level to better understand their performance. Except in the northeastern US, the eight-model ensembles show better agreement with the CASTNET O_3_ observations than the three boundary condition-model ensemble. Overall the three-model ensemble only outperforms one model but the eight-model ensemble outperforms seven individuals. This reflects that averaging the results from a larger number of models in this case more effectively canceled out the positive or negative biases from the individual models.

The monthly mean surface O_3_ from multiple global models’ free runs was also evaluated with the EANET observations. Among the three boundary condition models, GEOS-Chem produced higher O_3_ than the other two throughout the year, and C-IFS O_3_ is the lowest from April to December. The three-model and eight-model ensembles are lower than the surface O_3_ observations by <10 ppbv during high O_3_ seasons (winter/spring) but show substantial (> 10 ppbv) positive biases during low O_3_ seasons especially in July and August (Fig. 3b), similar to the HTAP1 model performance over Japan in 2001 (Fiore et al., 2009). During May–June 2010, generally the models performed better at the Japanese sites than at the South Korean sites (Table 2c), with significant positive biases occurring at low O_3_ regions (e.g., in central Japan) and negative biases found at high O_3_ regions, mainly owing to the uncertainty in the local and upwind emissions. The different approaches to generate the monthly mean modeled and the observed O_3_ data may have also contributed to these model-observation discrepancies. Overall O_3_ performance by individual models varies less significantly than at the CASTNET sites, with RMSEs ranging from 8.6 to ~13 ppbv (Table 2b). The three-model ensemble outperforms two individual models, and the eight-model ensemble outperforms six individual models. Unlike at the CASTNET sites, the three-model ensemble agrees better with the observations than the eight-model ensemble (Table 2c).

#### 3.1.3 Evaluation of the STEM regional base simulations with three sets of boundary conditions

The three STEM base simulations using different boundary conditions were evaluated with the hourly O_3_ observations at the CASTNET sites in the four US subregions. The evaluation included the 8 May–30 June 2010 period to exclude the results during the 1-week spin-up period. The time series plots of observed and modeled O_3_ at the western US CASTNET sites show that STEM was capable of capturing several high O_3_ periods, and it produced larger biases during the nighttime (Fig. 2c), as a result of the poorer WRF performance. Figure 2c and the evaluation statistics in Table 3a–b indicate that STEM/C-IFS O_3_ concentrations are associated with the highest positive bias and RMSE, while the STEM/GEOS-Chem and STEM/RAQMS predictions were positively and negatively biased by less than 2 ppbv, respectively, with similar RMSEs and correlations with the observations. The quality of the three STEM simulation means is closest to the STEM/GEOS-Chem run, with the mean bias/RMSE of ~1.6/4.9 ppbv, which is much better than the three-boundary model ensemble (−5.7/10.4 ppbv). However, this good performance can be a net effect of incorrect partitioning between the trans-boundary and local source contributions, with the former being underestimated and offsetting the overestimation of the latter. Switching the STEM chemical boundary conditions to the assimilated RAQMS base simulation led to increases in the simulated surface O_3_ concentrations by > 9 ppbv in the western US (Fig. S2, right), associated with higher positive biases (due to several factors discussed in the next paragraph). Regional-scale assimilation could further reduce uncertainties introduced from regional meteorological and emission inputs to obtain better modeled total O_3_ and the partitioning of trans-boundary versus US contributions (e.g., Huang et al., 2015).

The three STEM base simulations all significantly over-predicted O_3_ over the rest of the US in part due to the uncertainties in NO_*x*_ emissions, with STEM/RAQMS associated with the lowest RMSEs and mean biases, but STEM/C-IFS correlated best with the observations (Table 3b). These positive biases are higher than the global model ensembles’, which can partially result from the possible unrealistic VOC speciation of the emission inventory and the SAPRC 99 chemical mechanism. Although SAPRC mechanisms have been used in air quality modeling for regulatory applications in some US states such as California, they usually produced higher O_3_ than other mechanisms such as the CB04 and the CB05 (which were used by some HTAP2 global models, see Table 1c) over the US, and the comparisons between SAPRC 99 and SAPRC 2007 are still in progress (e.g., Luecken et al., 2008; Zhang et al., 2012; Cai et al., 2011). It is important to timely update the chemical mechanisms in the chemistry models, and we also suggest the timely upgrade of the VOC speciation in the bottom-up emission inventories in the US to benefit the air quality modeling. Additionally, the uncertainty from non-anthropogenic emissions, such as the biogenic VOC emissions from WRF/MEGAN, which is known to often have positive biases, can be another cause. As Hogrefe et al. (2011) presented, the MEGAN emissions resulted in a higher O_3_ response to hypothetical anthropogenic NO_*x*_ emission reductions compared with another set of biogenic emission input. Huang et al. (2017) showed that MEGAN’s positive biases are in part due to the positively biased temperature and radiation in WRF, and reducing ~2 °C in WRF’s temperature biases using a different land initialization approach led to ~20% decreases in MEGAN’s isoprene emission estimates in September 2013 over some southeastern US regions. These temperature and radiation biases, can also be important sources of uncertainty in the modeled O_3_ production. Quantifying the impacts of overestimated biogenic emissions and the biased weather fields that contributed to the biases in emissions on the modeled O_3_ is still an ongoing work. Some existing studies also reported O_3_ and NO_2_ biases from other regional models in the eastern US, due to the chemical mechanism and biases in NO_*x*_ and biogenic VOC emissions (e.g., Canty et al., 2015). We anticipate that the results from the Air Quality Model Evaluation International Initiative experiment (e.g., Schere et al., 2012; Solazzo et al., 2012; Galmarini et al., 2015, 2017), which involves more regional model simulations over the US with the similar set of boundary conditions but different chemical mechanisms and non-anthropogenic emission inputs, can help better understand the causes of errors in the simulated total O_3_.

### 3.2 The NAM surface O_3_ sensitivity to extra-regional anthropogenic pollutants

#### 3.2.1 Global model ensembles

**Figure 5:**
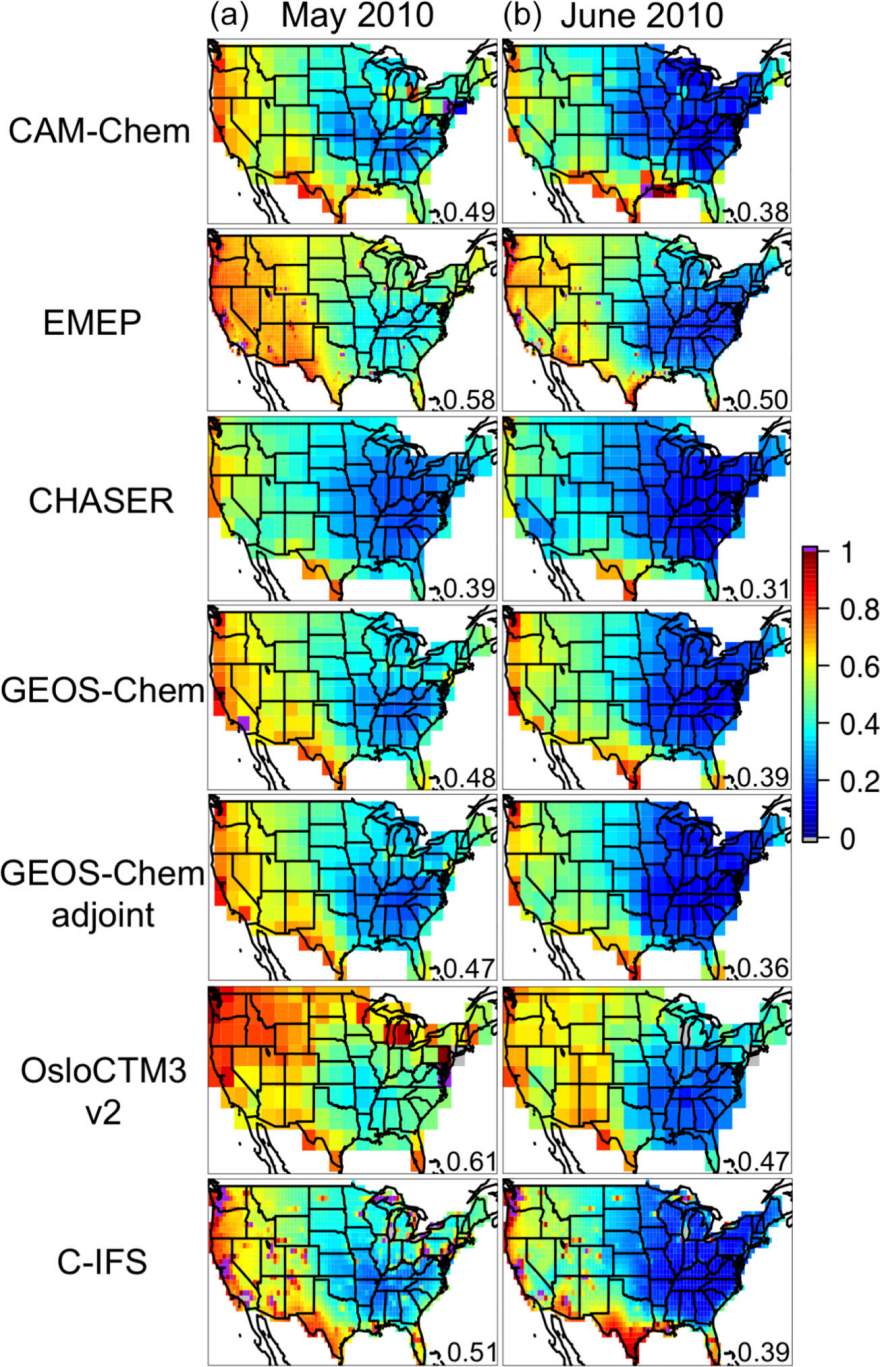
The RERER maps in (**a**) May and (**b**) June 2010 over the continental US, calculated based on the monthly mean O_3_ from multiple global models’ base and emission sensitivity simulations. The RERER metric (unitless) was defined in Eq. (2) in the text. Values larger than 1 and smaller than 0 are shown in purple and gray, respectively. The US (including continental US as well as Hawaii, which is not shown in the plots) mean values are indicated for each panel at the lower right corner. All models show declining RERER values from May to June, and the seven-model mean RERER values for May and June 2010 are ~0.5 and ~0.4, respectively.

**Figure 6:**
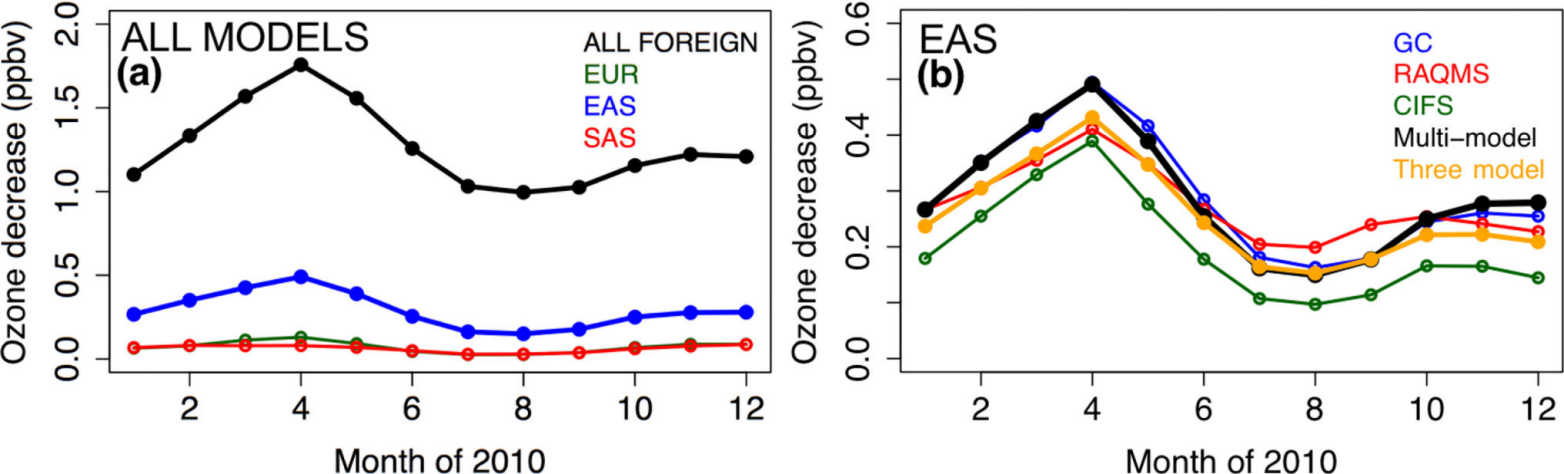
(**a**) North American (130–65° W; 20–50° N) mean O_3_ sensitivity to 20% anthropogenic emission reductions in various non-North American regions, averaged from multiple (six–eight, see details in text) global models. (**b**) North American surface *R*(O_3_, EAS, 20 %) values, as estimated by single (the three STEM boundary condition models) or multi-global model means. The “Multi-model” and “Three model” in the legend indicate the mean sensitivities of all eight global models and only of the three boundary condition models, respectively.

The impact of all foreign (i.e., non-NAM) anthropogenic sources on NAM surface O_3_ was first explored, including the spatial distributions of the RERER metric (Eq. 2) based on various global models’ simulations (Fig. 5) and the domain-wide mean sensitivities *R*(O_3_, non-NAM, 20 %) (Eq. 1d; Fig. 6). Across NAM, the strongest impacts were found in spring time (March–April–May, larger than 1.5 ppbv in average over the domain), and the weakest impacts are shown during the summertime (June–July–August, 1.0–1.3 ppbv), consistent with the existing knowledge on the seasonal variability of the non-local pollution impacts on NAM for other years (e.g., Fiore et al., 2009; Reidmiller et al., 2009). All global models indicate strong non-NAM anthropogenic source impacts on the western US mainly due to the impact of its high elevation, and also near the US-Mexico border areas, especially southern Texas, due to their vicinity to the Mexican (not included in the NAM source regions, see Fig. 1) emission sources. Over the western states, stronger non-local impacts were reflected from the results based on higher-horizontal-resolution global models (e.g., the >0.6 RERER values from the half degree EMEP model, corresponding to its higher *R*(O_3_, non-NAM, 20 %) values than the other models’), similar to the findings in previous modeling studies (Lin et al., 2010, 2012a). Although on a coarse horizontal resolution of 2.8°, OsloCTM3 suggests stronger extra-regional source influences on the northwestern US and the US-Canada border regions than the other models. Its largest number of vertical layers among all global models might be a cause. Larger-than-1 RERER values are often seen near the urban areas and large point sources due to the titration, especially evident from the higher-resolution model results. The *R*(O_3_, EAS, 20 %) values are larger than 1/3 of the *R*(O_3_, non-NAM, 20 %), 0.2–0.5 ppbv from April to June, more than 3–4 times higher than *R*(O_3_, EUR, 20 %) and *R*(O_3_, SAS, 20 %). Note that all eight models contributed to the *R*(O_3_, EAS, 20 %) calculations, but one or two models did not provide all necessary sensitivity runs to compute RERER, *R*(O_3_, non-NAM, 20 %), *R*(O_3_, EUR, 20 %), or *R*(O_3_, SAS, 20 %).

Comparing to the HTAP1 modeling results, the magnitudes of *R*(O_3_, EUR, 20 %) from this study are smaller by a factor of 2–3; in contrast, the *R*(O_3_, non-NAM, 20 %) and *R*(O_3_, EAS, 20 %) values are >50% higher than the HTAP1 modeling results. The different HTAP1 and HTAP2 results are possibly due to the following three reasons. First, the substantial improvement in the European air quality over the past decades that is shown in Crippa et al. (2016) and Pouliot et al. (2015), which contrasts with the growing anthropogenic emissions from East Asia and other developing countries during 2001–2010. Second, the changes in the HTAP2 experiment setup from HTAP1. This includes the differences in the participating models and the different region definitions, e.g., EUR by HTAP1’s definition includes regions in Russia, Belarussia, and Ukraine, the Middle East, and North Africa that are excluded from the HTAP2’s EUR domain. For EAS and SAS, however, the regions not overlapped by HTAP1 and HTAP2 are mostly in the less populated/polluted regions. Third, the stronger trans-Pacific transport in 2010 than in 2000–2001, as first introduced in Sect. 2.2.1. Interannual variability of *R*(O_3_, EAS, 20 %) and *R*(O_3_, non-NAM, 20 %) is also found between 2010 and 2008–2009, based on the SNU–GEOS-Chem calculations (Fig. S3). Foreign anthropogenic pollution impact on NAM was stronger in 2010 than in 2008–2009, especially in April–May. This can be in part due to the higher O_3_ precursors’ emissions in 2010 from extra-regions including East Asia (Table S1) as well as the spring 2010 meteorological conditions that favored the trans-Pacific pollution transport.

These monthly and regional-mean *R*(O_3_, EAS, 20 %) values suggest that despite dilution along the great transport distance, the EAS anthropogenic sources still had a distinguishable impact on the NAM surface O_3_. Similar to the findings from the HTAP1 studies, the large intermodel variability (as indicated in Table 4) in the estimates of intercontinental SR relationships indicates the uncertainties of these models in representing the key atmospheric processes which needs more investigations in the future. Figure 6b compares the *R*(O_3_, EAS, 20 %) values estimated by individual boundary condition models, their ensemble mean sensitivities, and the eight-global-model mean. The averaged *R*(O_3_, EAS, 20%) from the boundary condition model results are smaller than the eight-global-model mean, and, except for July–October 2010, GEOS-Chem gives higher *R*(O_3_, EAS, 20 %) than RAQMS and C-IFS, consistent with its highest O_3_ prediction in the EAS source region (Fig. 3b). Overall, *R*(O_3_, EAS, 20 %) and its intermodel differences are much smaller than the biases of the modeled total O_3_ in NAM. Other factors can contribute more significantly to the biases in the modeled total O_3_, such as the stratospheric O_3_ intrusion and the local O_3_ formation, and assessing the impacts from these factors would be also helpful for understanding the uncertainties in the modeled O_3_.

The O_3_ sensitivities in response to the perturbations of individual species or sector emissions in East Asia, estimated by the GEOS-Chem adjoint model, were also analyzed (Fig. S3). These sensitivities show similar seasonal variability to *R*(O_3_, EAS, 20 %), with the values ~twice as high in the spring than in summer, also consistent with the results on previous years based on the 20% emission perturbation approach (e.g., Fiore et al., 2009; Brown-Steiner and Hess, 2011; Emmons et al., 2012). However, this seasonal variability is weaker than the results based on the tagged tracer approach for earlier years. Using the CAM-Chem model, Brown-Steiner and Hess (2011) reported that during the springtime, Asian O_3_ created from the anthropogenic and biofuel NO_*x*_ emissions affected NAM O_3_ ~three times as strongly as in summer. This is because the nonlinear O_3_ chemistry, which is stronger outside of summer, caused larger O_3_ responses to a 100% reduction of NO_*x*_ emissions than 5 times of the O_3_ responses to a 20% reduction of NO_*x*_ emissions. The EAS anthropogenic NO_*x*_ emissions more strongly impacted the NAM surface O_3_ than the other major O_3_ precursors, similar to the findings in Fiore et al. (2009) and Reidmiller et al. (2009) using the perturbation approach, as well as the conclusions in Lapina et al. (2014) based on the adjoint sensitivity analyses. Emissions from the power and industrial sectors are higher in East Asia than in the other sectors (Table S1), resulting in its stronger influences on the NAM surface O_3_. As the observed NO_2_ columns started to drop since 2010 due to the effective denitration devices implemented at the Chinese power and industrial plants (e.g., Liu et al., 2016), depending on the changes in the VOC emissions, different *R*(O_3_, EAS, 20 %) values for the years after 2010 are anticipated. Therefore, continued studies to assess the East Asian anthropogenic pollution impacts on NAM during more recent years is needed. As emissions from various source sectors can differ by their emitted altitudes and temporal (from diurnal to seasonal) profiles, an effort should also be made to have the models timely update the heights and temporal profiles of the emissions from those various sectors.

#### 3.2.2 Regional model sensitivities and their connections with the boundary condition models’ sensitivities

**Figure 7:**
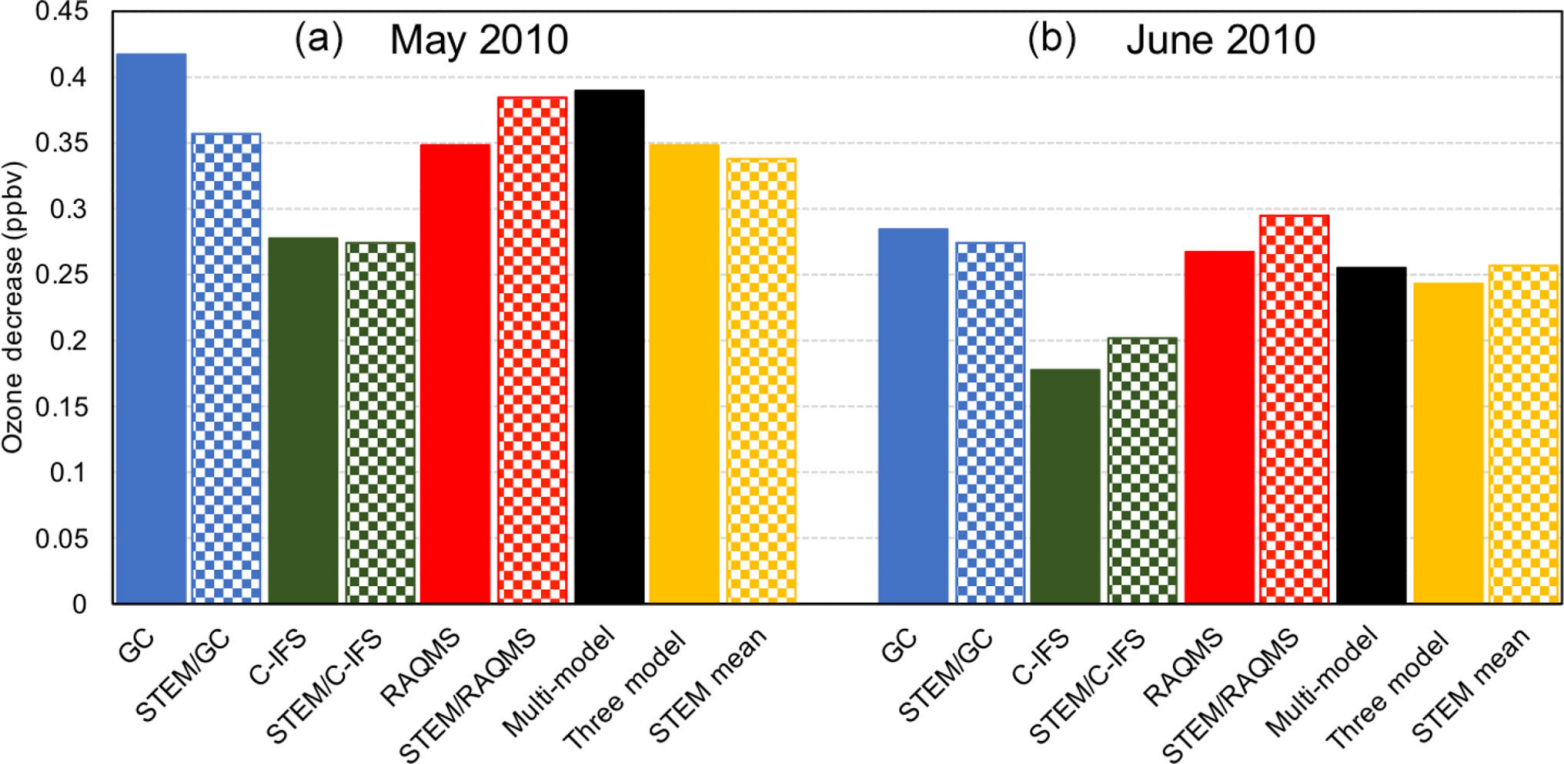
Monthly mean North American (130–65° W; 20–50° N) surface *R*(O_3_, EAS, 20 %) values from multiple global and regional model simulations for (**a**) May and (**b**) June 2010. STEM model mean values were calculated from its hourly output from 8 May and on. The “Multi-model” and “Three model” in the legend indicate the mean sensitivities of all eight global models and only of the three boundary condition models, respectively.

The monthly mean STEM surface *R*(O_3_, EAS, 20 %) sensitivities based on different boundary condition models were intercompared and also compared with the *R*(O_3_, EAS, 20 %) values estimated by their boundary condition models as well as the global model ensemble mean (Fig. 7). For both May and June 2010, the domain-wide mean *R*(O_3_, EAS, 20 %) values from STEM/RAQMS were higher than the estimates from RAQMS by 0.03 ppbv; the STEM/GEOS-Chem *R*(O_3_, EAS, 20 %) values are lower than those of GEOS-Chem by 0.01–0.06 ppbv, and the STEM/C-IFS *R*(O_3_, EAS, 20 %) is 0.02 ppbv higher than C-IFS’s in June but slightly (≪0.01 ppbv) lower in May. These differences are overall smaller than the inter-global model differences, and can be due to various factors including the uncertainties in boundary condition chemical species mapping, and the different meteorological, terrain fields, and chemistry in the global and regional model pairs. The STEM *R*(O_3_, EAS, 20 %) ensemble mean values, however, are less than 0.02 ppbv different from its boundary condition model’s ensemble mean for both months. The STEM *R*(O_3_, EAS, 20 %) ensemble mean value in June is also close to the eight-global-model-ensemble mean but is ~0.05 ppbv lower than the eight-model mean in May. Choosing other/more global model outputs as STEM’s boundary conditions may lead to different STEM ensemble mean *R*(O_3_, EAS, 20 %) estimates. We also found that the period-mean *R*(O_3_, EAS, 20 %) values of ~0.2 ppbv sampled only at the CASTNET sites (Table 3a) are smaller than those averaged in all model grids. This indicates that currently the sparsely distributed surface network (especially over the western US, which is more strongly affected by the extra-regional sources than the other US regions) may miss many LRT episodes that impact NAM. The planned geostationary satellites with ~2–5 km footprint sizes and hourly sampling frequency (Hilsenrath and Chance, 2013; Zoogman et al., 2017) will help better capture the high O_3_ and LRT episodes in these regions.

**Figure 8:**
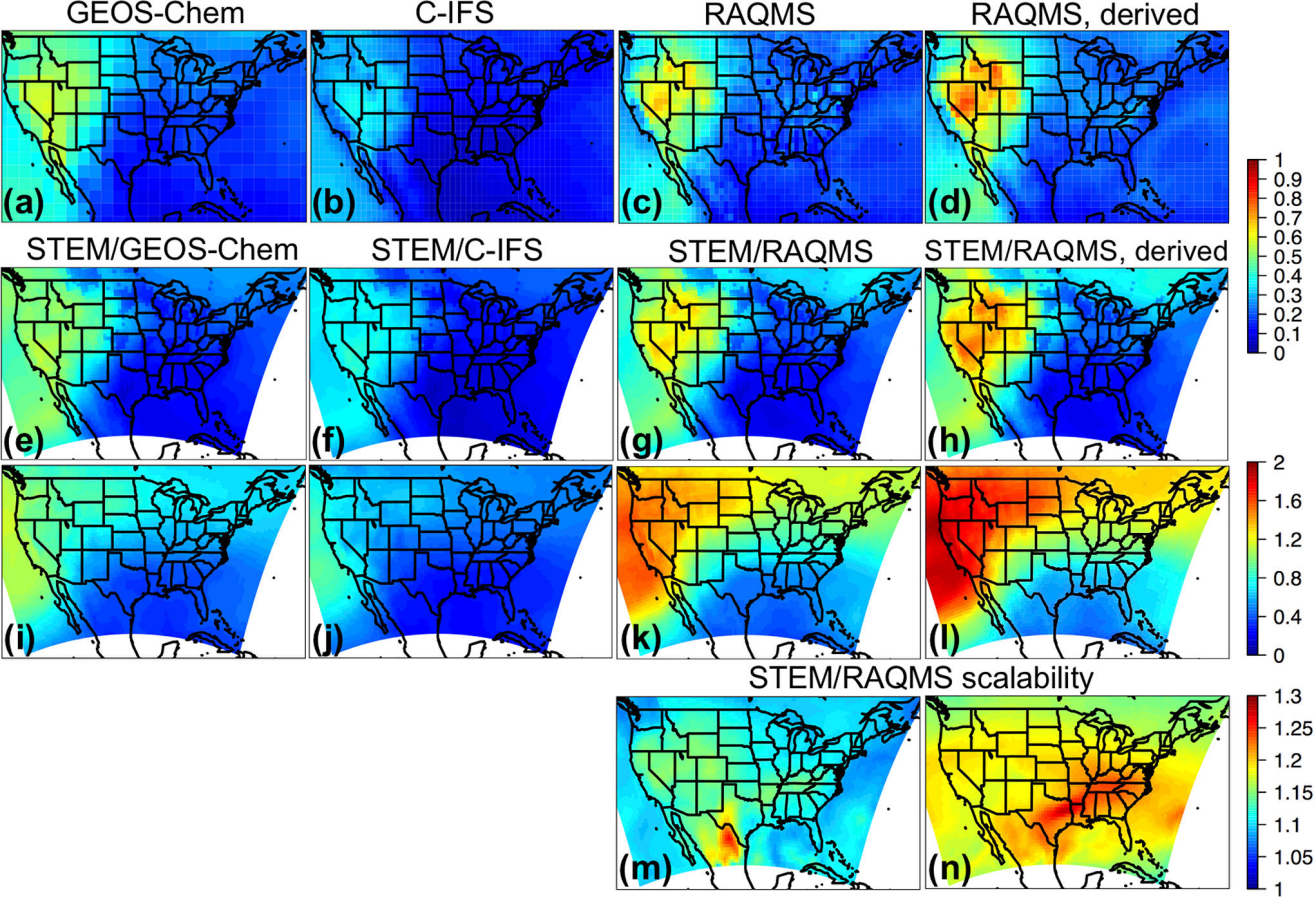
The monthly mean *R*(O_3_, EAS, 20 %) in June 2010 for (**a–d**) surface O_3_ (ppbv) from the three boundary condition models, (**e–h**) STEM surface O_3_ (ppbv), and (**i–l**) STEM column O_3_ (×10^16^ molecules cm^−2^). *R*(O_3_, EAS, 20 %) values from the simulations associated with GEOS-Chem, ECMWF C-IFS, and RAQMS are shown in (**a, e, i**), (**b, f, j**), and (**c, g, k**), respectively. Panels (**d, h, l**) show 1/5 of the *R*(O_3_, EAS, 100 %) values from the simulations related to RAQMS. STEM/RAQMS-based “scalability” *S*_O_3__ (Eq. 3) values over NAM are shown for (**m**) surface and (**n**) column O_3_.

**Figure 9:**
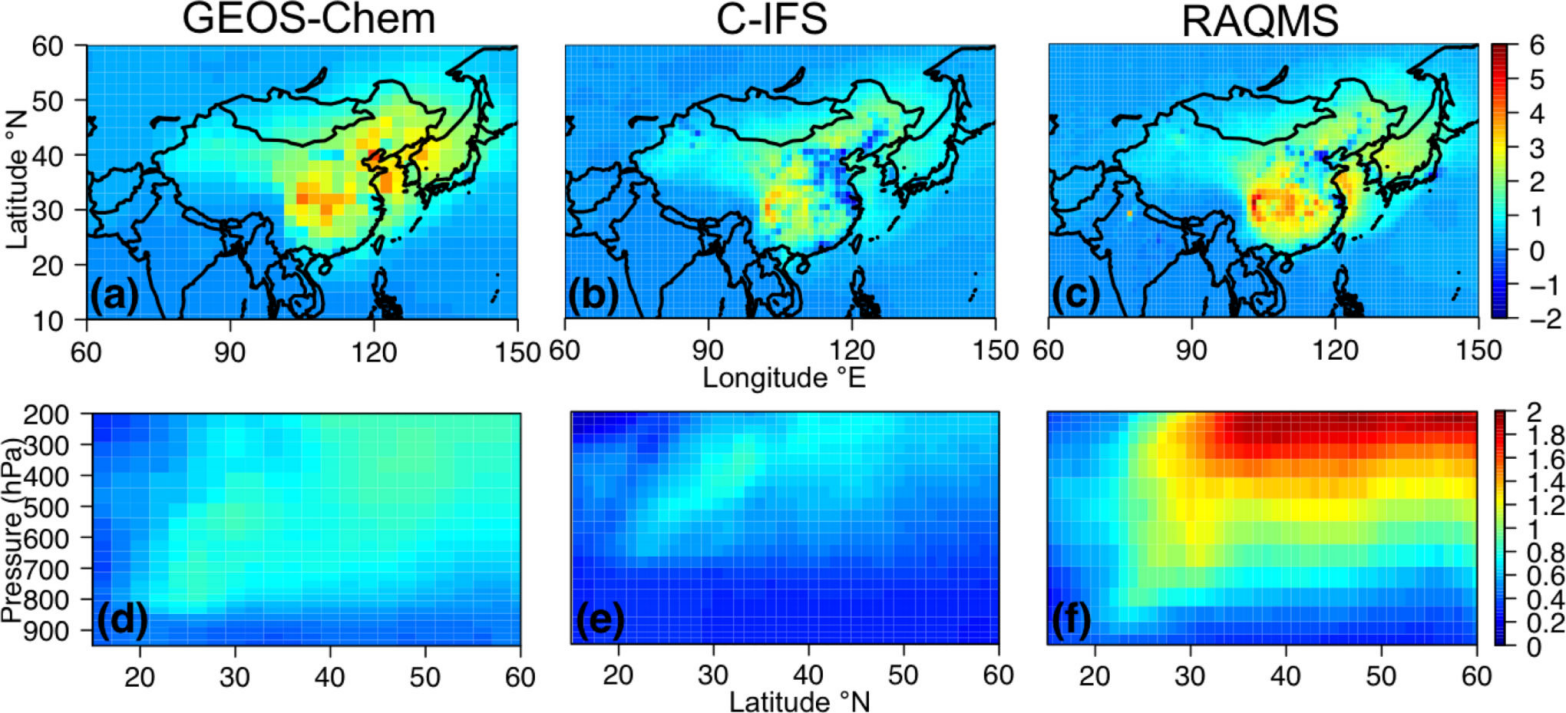
The monthly mean *R*(O_3_, EAS, 20 %) in ppbv in June 2010 from the three boundary condition models at the source and near the receptor regions: (**a–c**) surface O_3_ in East Asia; and (**d**) O_*x*_ (GEOS-Chem) or (**e–f**) O_3_ (ECMWF C-IFS and RAQMS) along the cross section of 135° W (near the west boundary of the STEM model domain as defined in Fig. 2a).

The spatial patterns of the monthly mean STEM surface *R*(O_3_, EAS, 20 %) sensitivities based on the three boundary condition models are notably different but overall resemble what is estimated by the corresponding boundary condition model, and the STEM sensitivities show more local details in certain high-elevation regions in the US west (Fig. 8 shows the June 2010 conditions as an example). These different sensitivities were investigated further by examining the *R*(O_3_, EAS, 20 %) values near the source regions (i.e., East Asia) as well as near the receptor regions (Fig. 9). More East Asian anthropogenic O_3_ seems to be transported at the upper troposphere in RAQMS than in the other two models. GEOS-Chem and RAQMS *R*(O_3_, EAS, 20 %) sensitivities are similar over EAS as well as the 500–900 hPa near the receptor in the eastern Pacific (at ~ 135 °W), which are the altitudes US surface O_3_ are most strongly sensitive to during the summertime as concluded from previous studies (e.g., Huang et al., 2010, 2013a; Parrish et al., 2010). Despite the close NAM domain-wide mean values from the STEM/GEOS-Chem and STEM/RAQMS, the spatial patterns of *R*(O_3_, EAS, 20 %) over NAM differ in these two cases, with the latter case showing sharper gradients especially in the western US, partially due to the impact of its higher horizontal resolution. The *R*(O_3_, EAS, 20 %) values from STEM/C-IFS are lower than from the other two cases both near the sources and at (near) NAM. The STEM surface (also near surface, not shown in figures) *R*(O_3_, EAS, 20 %) does not spatially correlate well with the column *R*(O_3_, EAS, 20 %), the latter of which contributed more to the base case O_3_ columns, indicating that a good portion of the transported East Asian pollution did not descend to the lower altitudes to impact the boundary layer/ground level air quality. An additional regional simulation was performed in which the STEM boundary conditions were downscaled from a RAQMS simulation without the East Asian anthropogenic emissions. The nonlinear emission perturbation–O_3_ response relationships, as the larger-than-1 *S*_O_3__ metric (Eq. 3) indicates, are seen across the domain, for both the surface and column O_3_ (Fig. 8). *S*_O_3__ values for column O_3_, ranging from 1.15–1.25 in most regions, are overall ~ 0.05 higher than *S*_O_3__ for the surface O_3_. Therefore, the full source contribution obtained by linearly scaling the receptor regional-mean O_3_ sensitivity to the 20% reduction in the source region emissions may be underestimated by at least ~ 10 %.

#### 3.2.3 Regional model MDA8 sensitivities on all days and during the O_3_ exceedances

The temporal variability of the STEM *R*(O_3_, EAS, 20 %) ensemble sensitivities were also studied. For most US subregions, 3–6 LRT episodes (defined as when the sensitivities are above the period mean) were identified during May–June. Only in certain regions, we find that the planetary boundary layer heights (PBLHs) were higher during the LRT episodes (i.e., the daily daytime-mean *R*(O_3_, EAS, 20 %) and PBLHs show medium-to-strong positive correlations (*r* > 0.5)), as these correlations may have been complicated by the relationships between the PBLHs and the local influences. Throughout this period, the hourly *R*(O_3_, EAS, 20 %) and the observed O_3_ at the surface CASTNET sites are weakly correlated (Table 3a), but they display similar diurnal cycles (e.g., Fig. 2c and d for the western US sites), possibly because the deeper boundary layer depth during the daytime enhanced entrainment down-mixing of the extra-regional pollutants to the surface. The identified diurnal variability of the *R*(O_3_, EAS, 20 %) values can cause differences in the calculated MDA8 and all-hour mean *R*(O_3_, EAS, 20 %) values. Figure S4 shows that the mean *R*(MDA8, EAS, 20 %) values, usually at daytime, are higher than the all-hour averaged *R*(O_3_, EAS, 20 %) in most STEM model grids during both months. Therefore, it is important for more HTAP2 participating models to save their outputs hourly in order to conveniently compute the policy-relevant metrics for the O_3_ sensitivities. Additionally, the hourly sampling frequency of the planned geostationary satellites is anticipated to be more helpful for evaluating the impacts of the LRT episodes.

**Figure 10:**
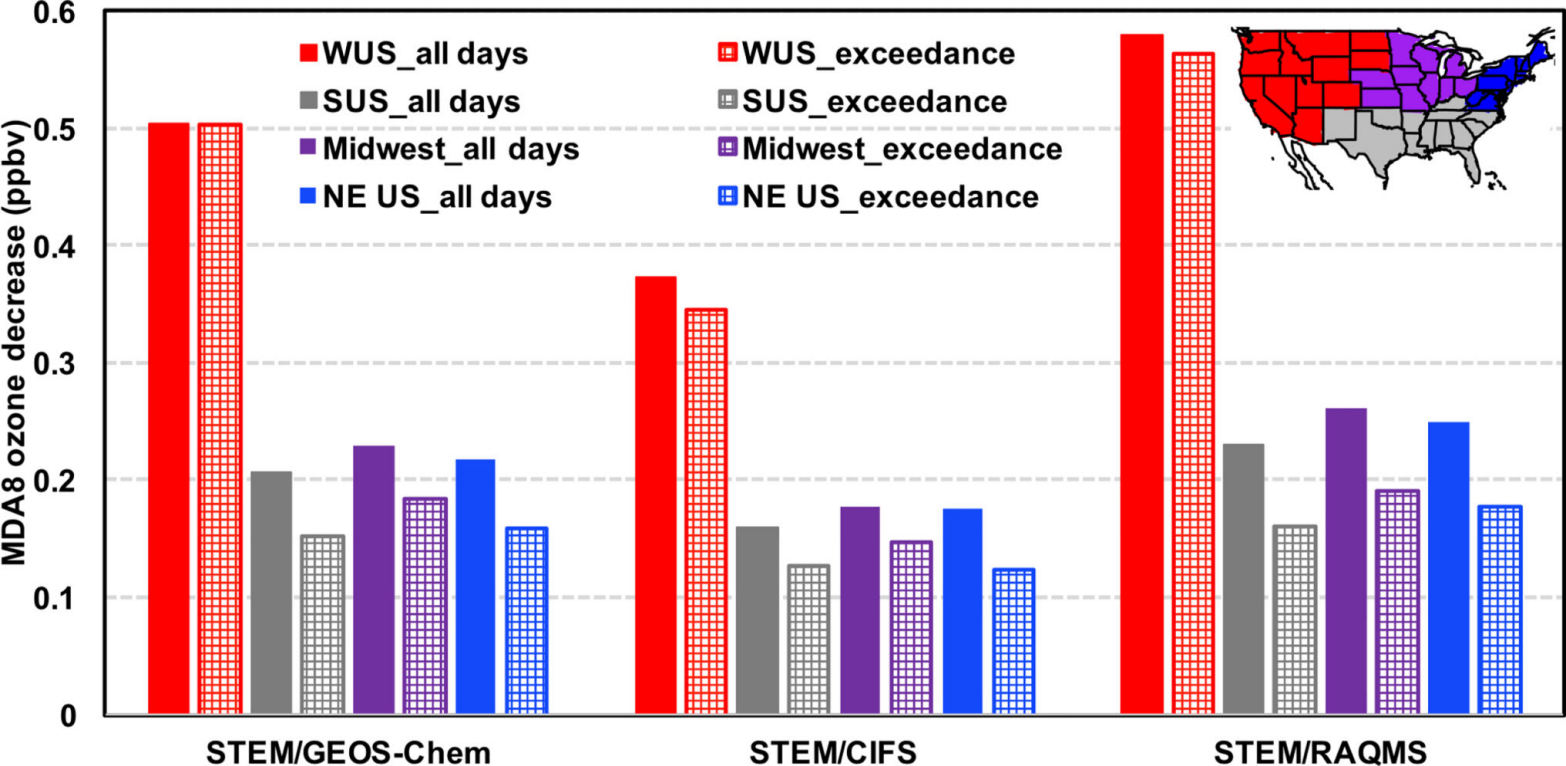
STEM *R*(MDA8, EAS, 20 %) for May–June 2010 in four US subregions (defined in the inset panel, also consistent with the definitions in Figs. 2 and S4 and Tables 2 and 3), averaged on all days (bars with solid fill) and only on the days when the simulated total MDA8 O_3_ concentrations were over 70 ppbv (bars with grid pattern fill). The results from the STEM runs using GEOS-Chem, ECMWF C-IFS, and RAQMS boundary conditions are shown separately.

The STEM *R*(MDA8, EAS, 20 %) in all model grids within the four US subregions were averaged on all days during May–June 2010 and only on the days when the simulated total MDA8 O_3_ is over 70 ppbv (Fig. 10). These sensitivities also show appreciable spatial variability: from 0.35–0.58 ppbv in the western US (also with the largest standard deviations, not shown), which is slightly higher than the HTAP1 results reported by Reidmiller et al. (2009) for Spring 2001, to ~0.1–0.25 ppbv in the remaining three subregions, which is close to the Reidmiller et al. (2009) results.

**Figure 11:**
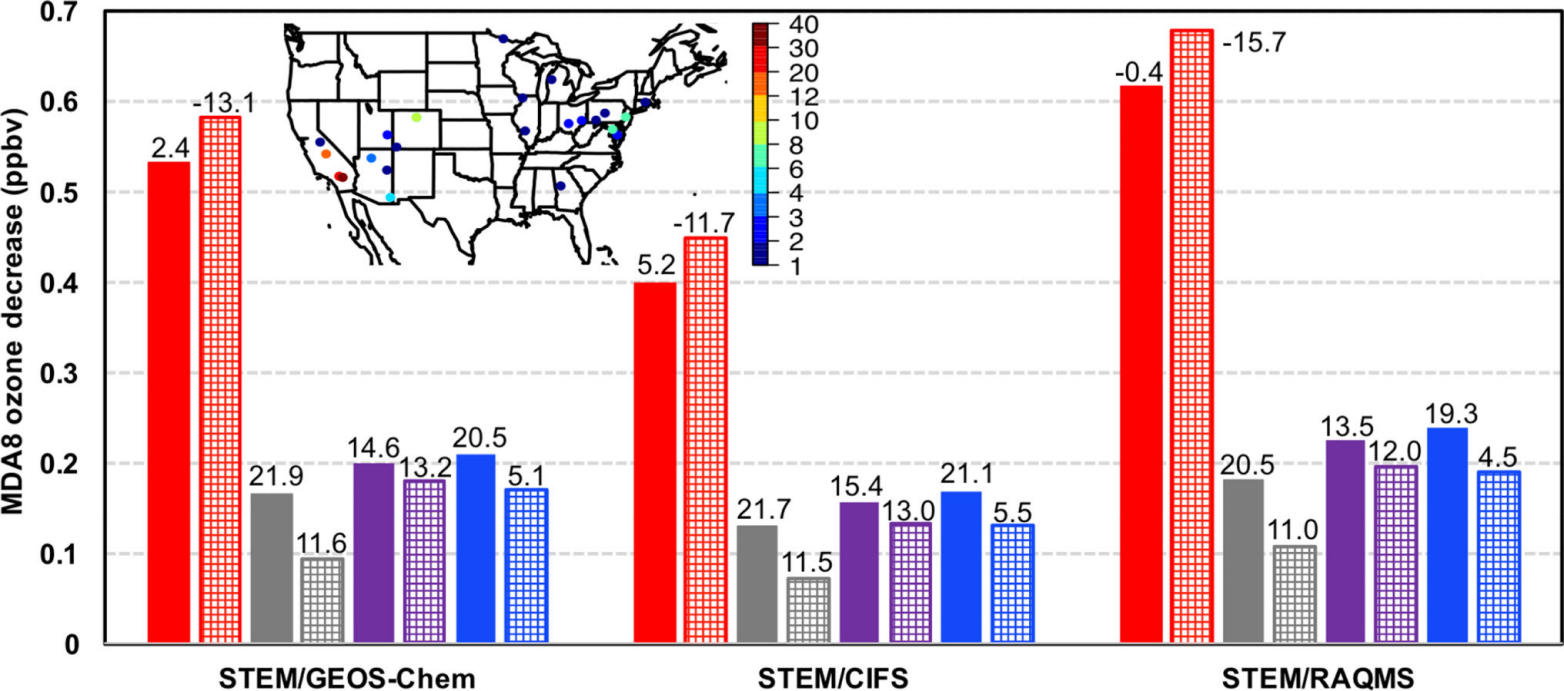
STEM *R*(MDA8, EAS, 20 %) for May–June 2010 at the CASTNET sites in four US subregions (same definition as in the Fig. 10 inset), averaged on all days (bars with solid fill) and only on the days when the observed MDA8 O_3_ concentrations were over 70 ppbv (bars with grid pattern fill). The results from the STEM runs using GEOS-Chem, ECMWF C-IFS, and RAQMS boundary conditions are shown separately. Biases for the corresponding model base runs are shown above the bar plots. Inset shows the number of days when the observed MDA8 O_3_ concentrations were over 70 ppbv at various CASTNET sites.

Comparing the solid bar plots in Figs. 10–11, we found that on all days in the three non-western subregions, *R*(MDA8, EAS, 20 %) values sampled at CASTNET sites are slightly smaller than those computed for all model grids, while in the non-western states the opposite differences are seen. This again suggests that expanding observation network would help better capture the high O_3_ and LRT episodes.

**Figure 12:**
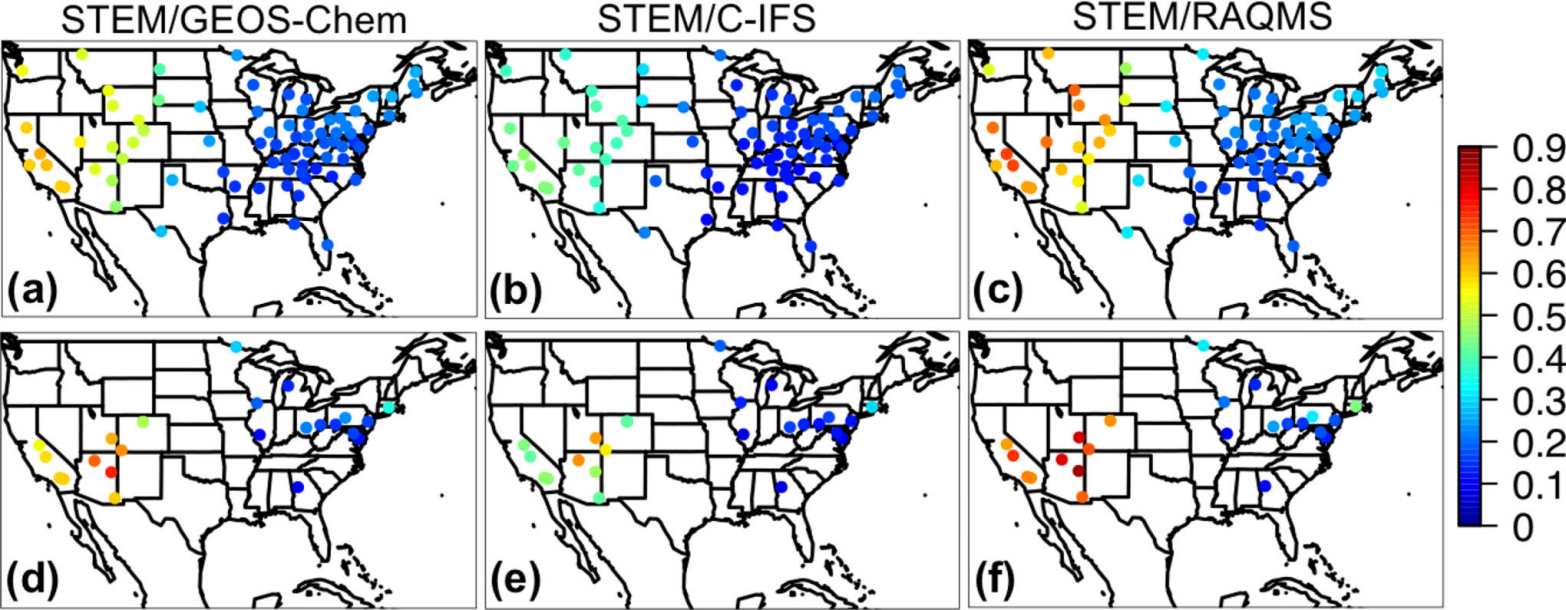
STEM *R*(MDA8, EAS, 20 %) in ppbv for May–June 2010 at the CASTNET sites on (**a–c**) all days and (**d–f**) the days when the observed MDA8 O_3_ concentrations were over 70 ppbv. The results from the STEM runs using (**a, d**) GEOS-Chem, (**b, e**) ECMWF C-IFS, and (**c, f**) RAQMS boundary conditions are shown separately.

Figure 10 suggests smaller *R*(MDA8, EAS, 20 %) values during the high O_3_ days in all subregions. However, STEM’s total O_3_ concentrations at CASTNET sites during the O_3_ exceedances were substantially overpredicted in non-western US regions while significantly underpredicted in the western US (see mean biases above the bar plots in Fig. 11). Therefore, the *R*(MDA8, EAS, 20 %) values shown in Fig. 10 during the model-based periods of O_3_ exceedances can represent the sensitivities during the actual periods of O_3_ compliance in non-western US regions and may not represent the sensitivities during all actual O_3_ exceedances in the western US. Figures 11–12 show that if calculated only at the CASTNET sites during the exceedances, in non-western US regions, *R*(MDA8, EAS, 20 %) is 0.02–0.07 ppbv smaller during the high O_3_ total days. This is qualitatively consistent with the findings in Reidmiller et al. (2009) and is possibly due to the fact that the LRT impacts were stronger on some days with good dispersion conditions when the NAAQS was not exceeded but weaker on some high O_3_ days under stagnant conditions. In contrast, western US *R*(MDA8, EAS, 20 %) at CASTNET sites was ~0.05 ppbv higher on high O_3_ days than for all days, and these differences are larger in rural/remote areas where local influences are less dominant. As a result, the medium-to-strong positive correlations are found between the modeled LRT of pollution and the total O_3_ in these regions (Table 3a; Lin et al., 2012a).

### 3.3 Case studies of spring (9 May) and summer (10 June) LRT events mixed with stratospheric O_3_ intrusions

**Figure 13:**
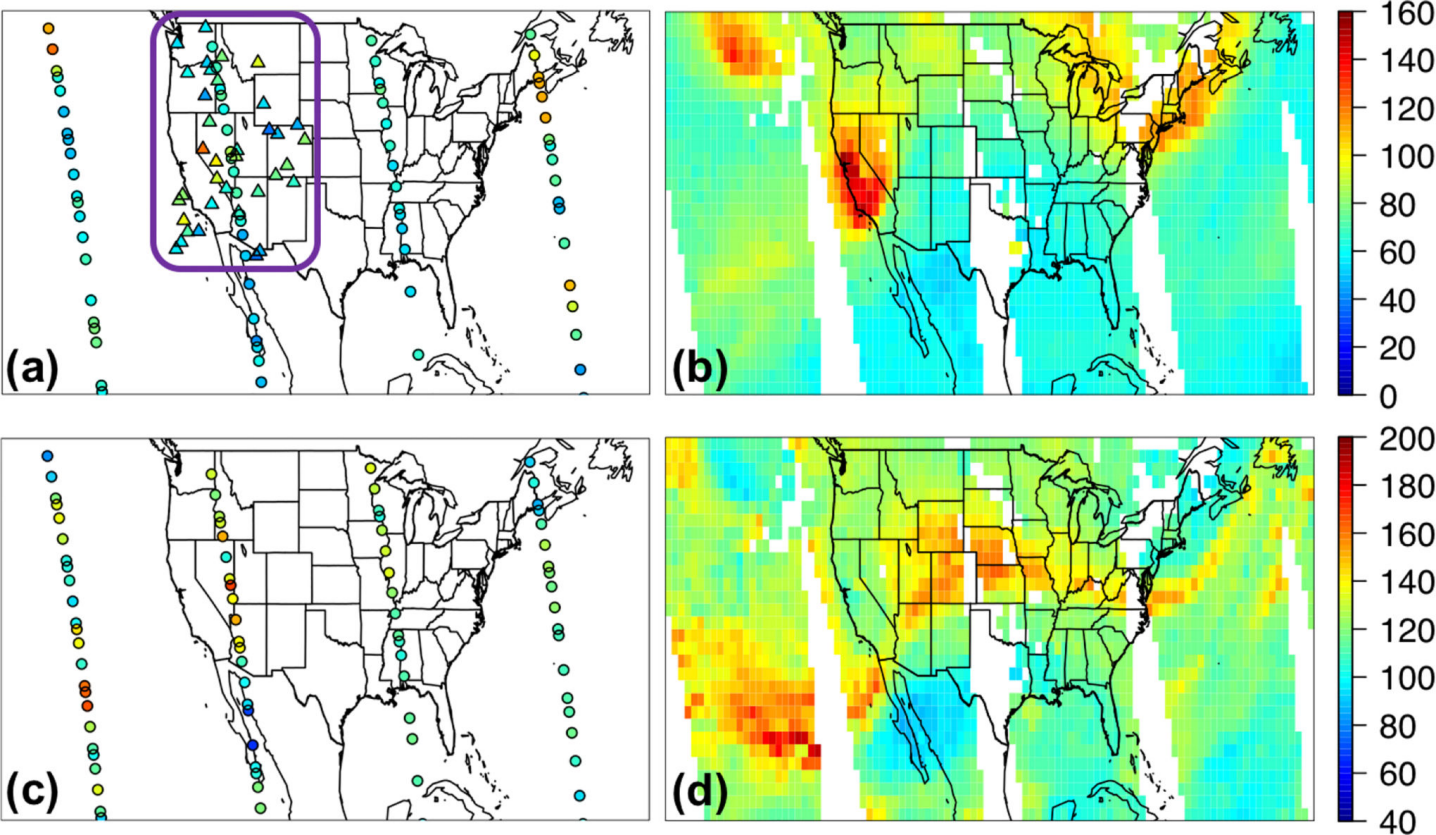
Case study of 9 May 2010: (**a–b**) Ozone (ppbv) and (**c–d**) CO (ppbv) at ~500 hPa from the L2 (**a, c**) TES retrievals (circles) and (**b, d**) L3 AIRS products at early afternoon local time. The L2 IASI O_3_ (ppbv) at ~500 hPa retrieved using the TES algorithm (details in Sect. 2.3.2) at the mid-morning local times is shown on (**b**) as triangles. The O_3_ profiles within the purple box in (**a**) were used in the model evaluation shown in Fig. 14.

Lin et al. (2012a, b) and Neuman et al. (2012) showed that the trans-Pacific pollution transport intensely impacted the western US during 8–10 May 2010, intermingled with a stratospheric intrusion that contributed to at least 1/3 of the total O_3_ in some high-elevation regions. This episode is indeed indicated by the O_3_ and CO products from AIRS and TES at ~500 hPa over the eastern Pacific (Fig. 13), and the observed TES and IASI O_3_ profiles over the western US indicated elevated O_3_ levels (>80 ppbv) at 700–900 hPa. Huang et al. (2013b) found that the meteorological conditions during this period (i.e., a strong jet at ~700 hPa with wind speed > 20 m s^−1^ shifted southwesterly when passing southern California and continued to travel towards the mountain states), along with the orographic lifting, efficiently exported the southern California anthropogenic pollution, which was chemically coupled with the extra-regional pollution and significantly enhanced the O_3_ levels in the US intermountain west.

**Figure 14:**
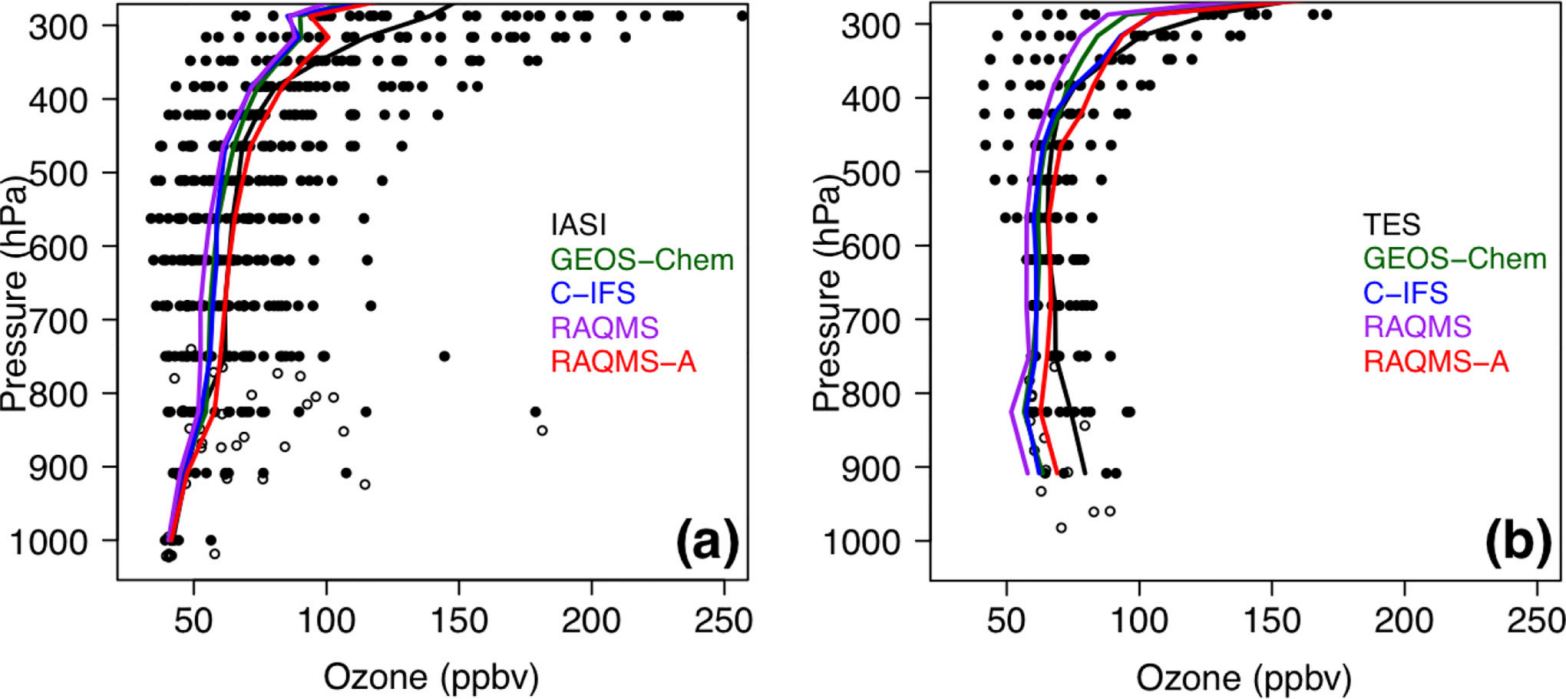
Case study of 9 May 2010: the comparisons between (**a**) IASI and (**b**) TES O_3_ in the western US with the simulated O_3_ in the STEM runs using the GEOS-Chem (green), C-IFS (blue), RAQMS (purple), and assimilated RAQMS (red) boundary conditions. The O_3_ profiles within the purple box in Fig. 13a were used in the evaluation. Observation operators were applied in the comparisons (details in Sect. 2.3.2). Solid and open dots are TES or IASI data at the TES retrieval reporting levels and at the variable surface pressure levels, respectively. Solid lines are median O_3_ profiles from the satellite observations and the different STEM simulations, calculated only on the TES retrieval reporting levels.

**Figure 15:**
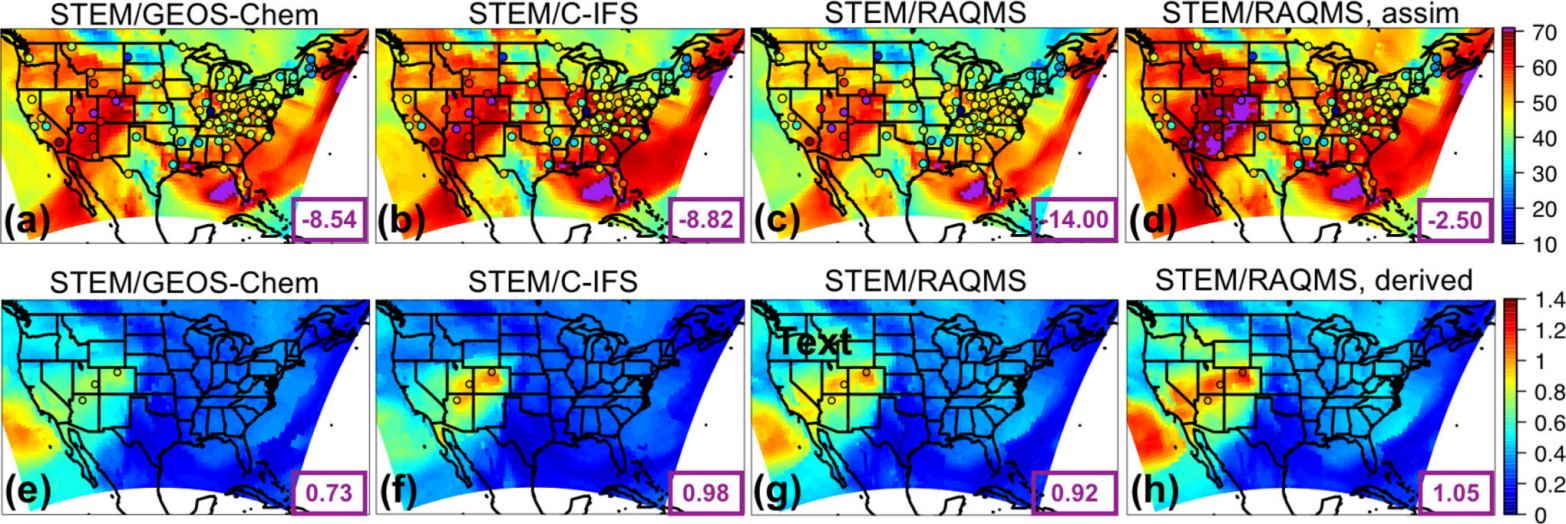
Case study of 9 May 2010: (**a–d**) Surface MDA8 total O_3_ and (**e–h**) surface *R*(MDA8, EAS, 20 %) from the STEM simulations using the (**a, e**) GEOS-Chem, (**b, f**) ECMWF C-IFS, and (**c, g**) RAQMS free run as the boundary conditions. (**d**) Surface MDA8 total O_3_ in a STEM base simulation using the RAQMS assimilation run as the boundary conditions. CASTNET observations are overlaid in filled circles in (**a–d**). (**h**) is 1/5 of the surface *R*(MDA8, EAS, 100 %) from STEM/RAQMS simulations. The conditions at ~400–500 hPa are shown in Fig. S5. Purple numbers at the lower right corners of (**a–d**) and (**e–h**) are mean model biases and mean *R*(MDA8, EAS, 20 %) values in ppbv at the three mountain sites (Grand Canyon NP, AZ; Canyonlands NP, UT; and Rocky Mountain NP, CO) where O_3_ exceedances were observed on this day. The locations of these sites are shown in (**e–h**) as open circles.

We selected this episode to compare the STEM surface total O_3_ concentrations as well as the *R*(O_3_, EAS, 20 %) sensitivities based on the different HTAP2 boundary condition models. Figure 14 evaluates the simulated O_3_ profiles in the western US from several STEM base simulations against the TES and IASI O_3_ retrievals, and Fig. 15a–d indicate the performance of the daily surface total MDA8 O_3_ from these simulations. We found that the underestimated free tropospheric O_3_ from the STEM simulations that used any single free-running chemical boundary conditions contributed to the underestimated STEM surface O_3_ in the high-elevation mountain states – e.g., by 9–14 ppbv at three CASTNET sites (Grand Canyon National Park [NP], AZ; Canyonlands NP, UT; and Rocky Mountain NP, CO) where O_3_ exceedances were observed. The unsatisfactory performance by free-running global models during high O_3_ events would pose difficulties for regional models (regardless of their resolutions and other configurations, parameterization) to accurately estimate the SR relationships using boundary conditions downscaled from these model runs. The STEM base simulation using the RAQMS assimilated fields as the boundary conditions agrees most with the observed O_3_ at the CASTNET sites as well as the TES and IASI O_3_ profiles in the western states. Similar to the conclusions drawn in Huang et al. (2010, 2015) for summer 2008, we again demonstrated the robustness of satellite chemical data assimilation for improving the boundary condition models’ O_3_ performance. As the enhancement of O_3_ due to the assimilation is much larger than the O_3_ sensitivities to the EAS anthropogenic emissions, the assimilation mainly improved the contributions from other sources, possibly including the stratospheric O_3_.

The quality of the model boundary conditions only indicates how well the total “transported background” component is represented and can not be directly connected with the accuracy of the model-estimated *R*(O_3_, EAS, 20 %) sensitivities, which also show notable intermodel differences. The estimated *R*(MDA8, EAS, 20 %) in the different STEM cases range from < 1.0 to ~1.3 ppbv, at least 40% higher than the May–June period mean in Figs. 10–11. The mean *R*(MDA8, EAS, 20 %) at three high O_3_ CASTNET sites ranges from 0.73 (STEM/GEOS-Chem) to 0.98 ppbv (STEM/C-IFS), with the mean *S*_O_3__ of ~1.14 at these sites based on the STEM/RAQMS runs due to the nonlinear emission perturbation–O_3_ response relationships (Fig. 15e–h). *R*(MDA8, EAS, 100 %) from the STEM/RAQMS case is as high as > 7 ppbv over the high-terrain regions. These are of smaller magnitudes than the estimates in Lin et al. (2012a), possibly due to the differences in the used models and the bottom-up emission inputs.

A stratospheric O_3_ intrusion also affected the northeastern US on the same day, as revealed by the satellite midtropospheric O_3_ and CO observations (Fig. 13). This intrusion was mixed with LRT East Asian pollution (Figs. 15 and S5). However, this intrusion did not enhance the northeastern US boundary layer/surface O_3_ concentrations, which were actually anomalously low (MDA8 < 40 ppbv), as indicated by the model base simulations and the CASTNET observations (Fig. 15a–d). Similar characteristics during summertime stratospheric O_3_ intrusion events around this region have been discussed by Ott et al. (2016). The East Asian pollution affected the surface O_3_ levels less intensely (< 50 %) in these regions than in the US west, due to the greater transport distances, stronger local emission influence on chemical production/loss, shallower PBLHs, and the impact of the overall flat terrain in the US east.

**Figure 16:**
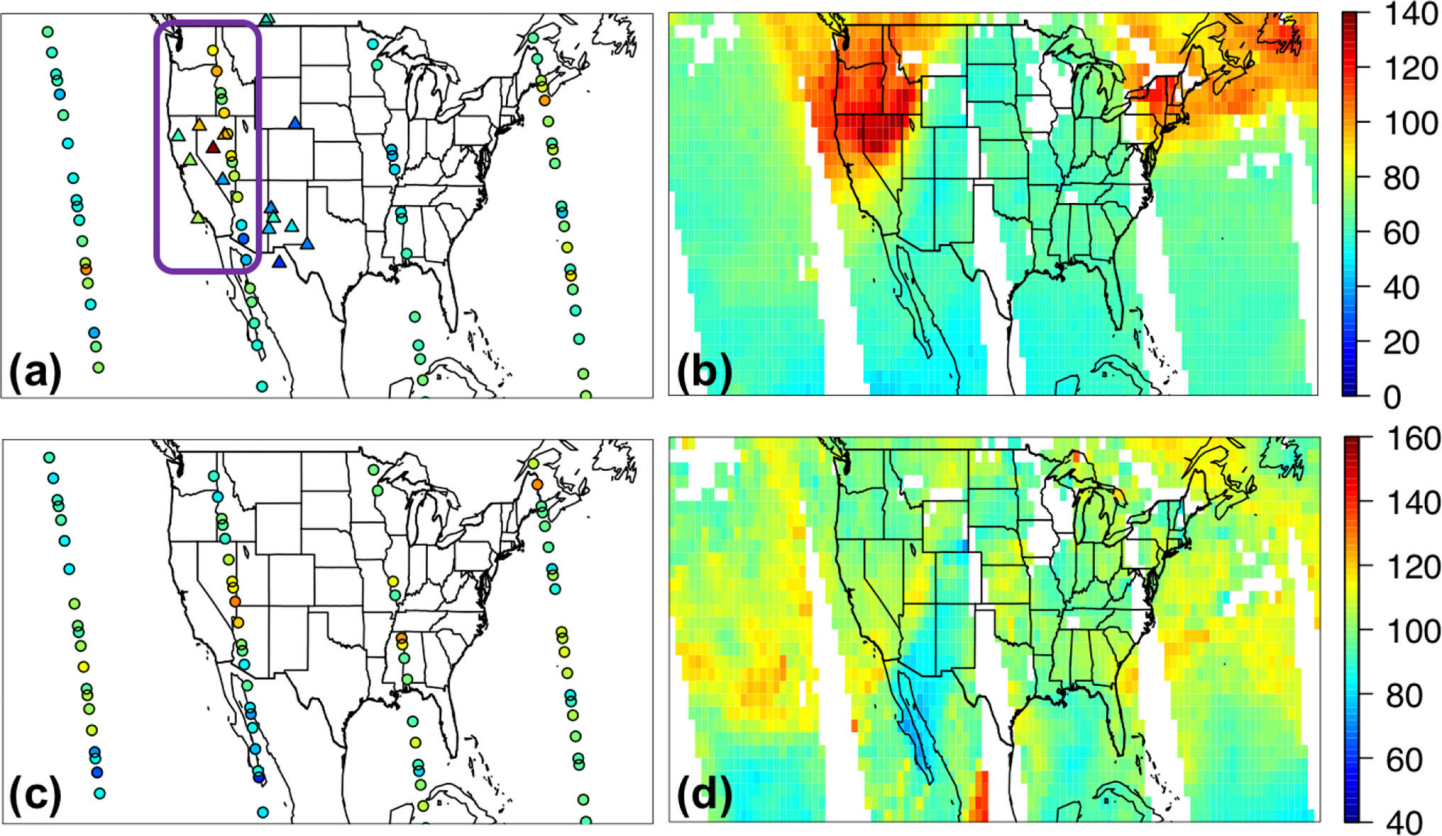
Same as Fig. 13, but for a case study of 10 June 2010.

**Figure 17:**
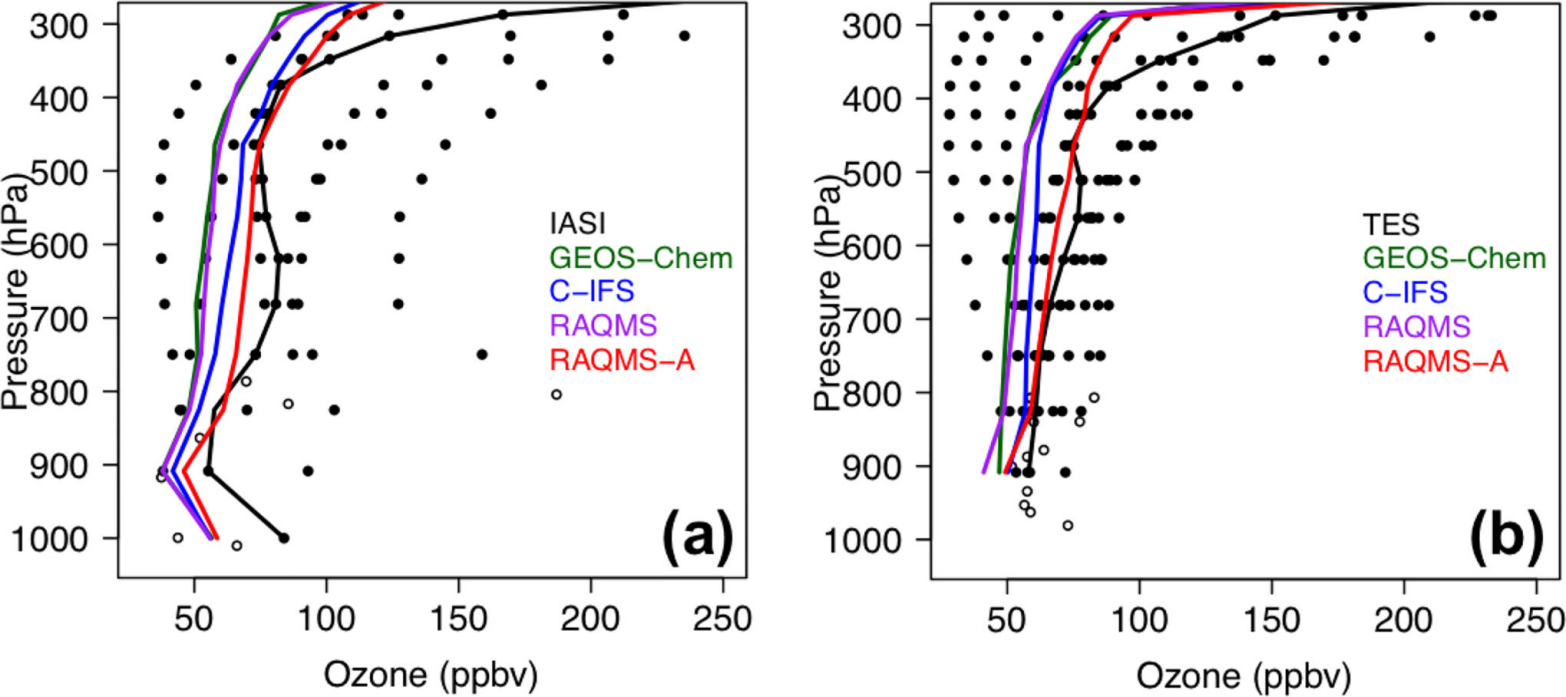
Same as Fig. 14, but for a case study of 10 June 2010.

**Figure 18:**
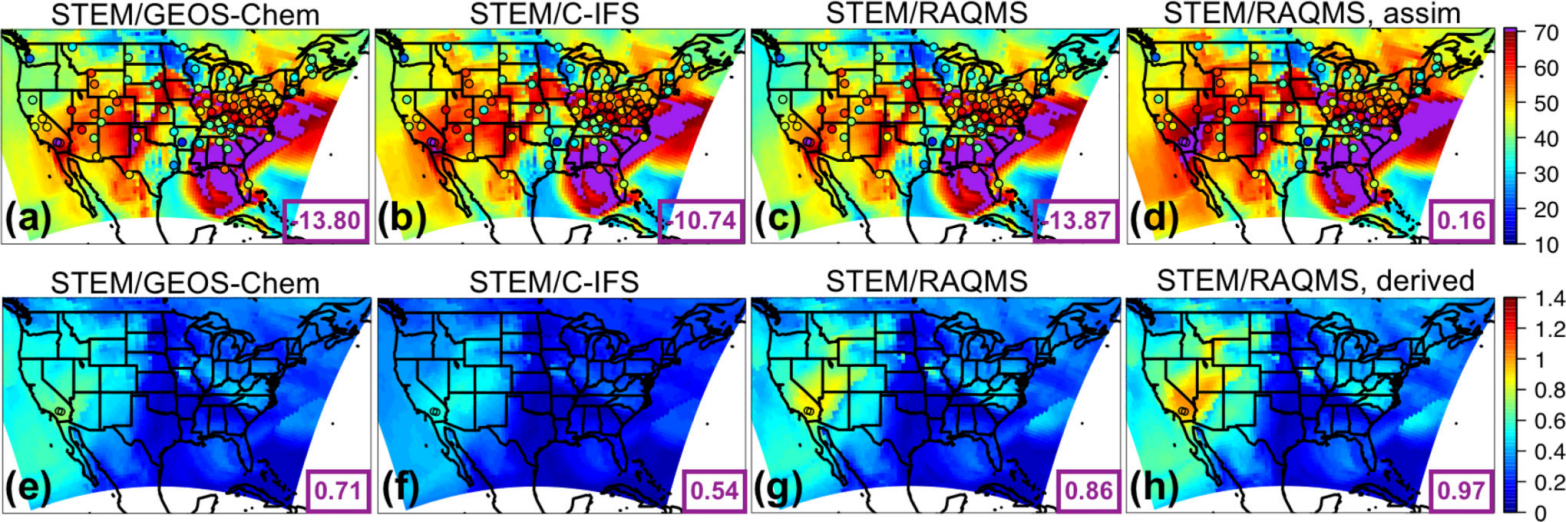
Same as Fig. 15, but for a case study of 10 June 2010. The CASTNET sites with O_3_ exceedances on this day are Converse Station and Joshua Tree NP in southern California.

A summertime LRT event on 9–10 June is analyzed to contrast with the 9 May case study. Lin et al. (2012b) showed that > 80 ppbv of ozonesonde data in northern California at 2–6 km measured the stratospheric O_3_ remnants during this episode, and the transported stratospheric O_3_ contributed to as much as ~50% of the total O_3_ in southern California based on their model calculations. We show that on 10 June over 100 ppbv of O_3_, as well as < 90 ppbv CO, was observed by satellites at ~500 hPa above Nevada and northern California (Fig. 16), which again was substantially underestimated by all free-running models (Fig. 17), resulting in the underpredicted total O_3_ at two CASTNET sites in southern California (Converse Station and Joshua Tree NP) that experienced O_3_ exceedances on this day (Fig. 18a–c). The negative biases in the transported background O_3_ and surface MDA8 O_3_ were successfully reduced by incorporating satellite data (Figs. 17 and 18d).

Figure 18e–h show that LRT of EAS anthropogenic pollution also strongly affected southern California and Nevada. Notable intermodel differences are again found in the estimated *R*(MDA8, EAS, 20 %), but they are overall lower than on 9 May (< 1.0 ppbv). The mean *R*(MDA8, EAS, 20 %) at the two high O_3_ CASTNET sites ranges from 0.54 (STEM/C-IFS) to 0.86 ppbv (STEM/RAQMS), with the mean *S*_O_3__ of ~1.13 at these sites based on the STEM/RAQMS runs (Fig. 18e–h). *R*(MDA8, EAS, 100 %) from the STEM/RAQMS case is as high as > 6 ppbv over southern California and Nevada. Compared to the spring event, *R*(MDA8, EAS, 20 %) in the eastern US are discernable only over a limited region, due to weaker transport and stronger local chemical production/loss.

## 4 Conclusions and suggestions on future directions

In support of the HTAP phase 2 experiment that involved high-resolution global models and regional models’ participation to advance the understanding of the pollutants’ SR relationships in the Northern Hemisphere, we conducted a number of regional-scale STEM base and forward sensitivity simulations over NAM during May–June 2010. STEM’s top and lateral chemical boundary conditions were downscaled from three global models’ (i.e., GEOS-Chem, RAQMS, and ECMWF C-IFS) base and sensitivity simulations (in which the East Asian anthropogenic emissions were reduced by 20 %). Despite dilution along the great transport distance, the East Asian anthropogenic sources still had a distinguishable impact on the NAM surface O_3_, with the period-mean NAM O_3_ sensitivities to a 20% reduction of the East Asian anthropogenic emissions, i.e., *R*(O_3_, EAS, 20 %), ranging from ~0.24 ppbv (STEM/C-IFS) to ~0.34 ppbv (STEM/RAQMS). The spatial patterns of the STEM surface O_3_ sensitivities over NAM overall resembled those from its corresponding boundary condition model, with regional/period-mean *R*(O_3_, EAS, 20 %) differing slightly (<10 %) from its corresponding boundary condition model, which are smaller than those among its boundary condition models. The boundary condition models’ 2-month mean *R*(O_3_, EAS, 20 %) was ~8% lower than the mean sensitivity estimated by eight global models. Therefore, choosing other global model outputs as STEM’s boundary conditions may lead to different STEM O_3_ sensitivities. The biases and RMSEs in the simulated total O_3_, which differed significantly among models, can partially be due to the uncertainty in the bottom-up NO_*x*_ emission inputs according to the model comparison with the OMI NO_2_ columns, and future work on attributing the intermodel differences on process level is particularly important for better understanding the sources of uncertainties in the modeled total O_3_ and its source contribution.

The HTAP2 multi-model ensemble mean *R*(O_3_, EAS, 20 %) values in 2010 were higher than the HTAP1 reported 2001 conditions, due to a number of reasons including the impacts of the growing East Asian anthropogenic emissions, the interannual variability in atmospheric circulation (i.e., stronger trans-Pacific transport in spring 2010 following an El Niño event), and the different experiment designs of HTAP1 and HTAP2. The GEOS-Chem O_3_ sensitivities in 2010 were also higher than the 2008–2009 conditions due to the increasing Asian emissions and the spring 2010 meteorological conditions that favored the trans-Pacific pollution transport. The GEOS-Chem sensitivity calculations indicate that the East Asian anthropogenic NO_*x*_ emissions mattered more than the other East Asian O_3_ precursors to the NAM O_3_, qualitatively consistent with previous adjoint sensitivity calculations. Continued research is needed on temporal changes of emissions for different species and sectors in NAM and foreign countries as well as their impacts on the SR relationships. As emissions from various source sectors can differ by emitted altitudes and temporal profiles, efforts should also be placed to have the models timely update the height and temporal profiles of the emissions from various sectors.

An additional STEM simulation was performed in which the boundary conditions were downscaled from a RAQMS simulation without East Asian anthropogenic emissions (i.e., a 100% emission reduction) to assess the scalability of the mean O_3_ sensitivities to the size of the emission perturbation. The scalability was found to be spatially varying, ranging from 1.15–1.25 for column O_3_ in most US regions, which were overall ~0.05 higher than in the surface O_3_. Therefore, the full source contribution obtained by linearly scaling the NAM regional-mean O_3_ sensitivity to the 20% reduction in the East Asian emissions may be underestimated by at least 10 %. The underestimation in other seasons of the HTAP2 study period may be higher and will need to be quantified in future work. Moreover, motivated by Lapina et al. (2014), additional calculations will be conducted in future studies to explore the scalability of different O_3_ metrics in these cases. For future source attribution analysis, it is generally recommended to directly choose the suitable size of the emission perturbation based on the specific questions to address and to avoid linearly scaling O_3_ sensitivities that are based on other amounts of the perturbations.

The STEM O_3_ sensitivities to the East Asian anthropogenic emissions (based on three boundary condition models separately and averagely) were strong during 3–6 episodes in May–June 2010, following similar diurnal cycles as the total O_3_. Stronger East Asian anthropogenic pollution impacts were estimated during the observed O_3_ exceedances in the western US than on all days, especially over the high-terrain rural/remote areas; in contrast, the East Asian anthropogenic pollution impacts were not as strong during O_3_ exceedances in other US regions. We emphasized the importance of saving model results hourly for conveniently calculating policy-relevant metrics as well as the usefulness of hourly sampling frequency of the planned geostationary satellites for better evaluating the impacts of the LRT events.

Based on model calculations, satellite O_3_ (TES, JPL-IASI, and AIRS), CO (TES and AIRS), and surface O_3_ observations on 9 May 2010, we showed the different influences from stratospheric O_3_ intrusions along with the transported East Asian pollution on O_3_ in the western and the eastern US. This event was further compared with a summer event on 10 June 2010. During both events, the unsatisfactory performance of free-running (i.e., without chemical data assimilation) global models would pose difficulties for regional models (regardless of their resolutions and other configurations, parameterization) for accurately simulating the surface O_3_ and its source contribution using boundary conditions downscaled from these model runs. Incorporating satellite (OMI and MLS) O_3_ data effectively improved the modeled O_3_. As chemical data assimilation techniques keep developing (Bocquet et al., 2015), several HTAP2 participating global models have already been able to assimilate single- or multi-constitute satellite atmospheric composition data (e.g., Miyazaki et al., 2012; Parrington et al., 2008, 2009; Huang et al., 2015; Inness et al., 2015; Flemming et al., 2017). Comparing the performance of the assimilated fields from different models and making the global model assimilated chemical fields in the suitable format for being used as boundary conditions would be very beneficial for future regional modeling as well as for better interpreting the pollutants’ distributions, especially during the exceptional events. Meanwhile, efforts should also be devoted to advancing and applying higher-resolution regional-scale modeling and chemical data assimilation. Furthermore, although satellite observations have been applied to improve the estimated US background O_3_ (e.g., Huang et al., 2015), the use of satellite (and/or other types of) observations to improve SR relationship studies also needs to be explored. Some of the possible methods include (1) the combination of data assimilation and the tagging approach and (2) the introduction of observation-constrained emission estimates in the emission perturbation analyses.

## Supplementary Material

Sup 1

## Figures and Tables

**Table 1:** (**a**) HTAP2 base and sensitivity simulations by various global models. (**b**) STEM regional simulations for HTAP2. (**c**) STEM and its boundary condition models’ key inputs and chemical mechanisms, with references. More details on the models can be found in (**a**) and the text.

(a) Global model, resolution: lon × lat × vertical layer, (References)	BASE	EASALL (−20 %)	EASALL (−100 %)	GLOALL (−20 %)	NAMALL (−20 %)	EURALL (−20 %)	SASALL (−20 %)
CAM-Chem, 2.5° × 1.9° ×56 (Tilmes et al., 2016)	✓	✓		✓	✓	✓	✓
CHASER T42, ~2.8° × 2.8° × 32 (Sudo et al., 2002)	✓	✓		✓	✓	✓	✓
EMEP rv48, 0.5° × 0.5° × 20 (Simpson et al., 2012)	✓	✓		✓	✓	✓	✓
SNU–GEOS-Chem v9-01-03, 2.5° × 2° × 47 (Park et al., 2004; http://iek8wikis.iek.fz-juelich.de/HTAPWiki/WP2.3?action=AttachFile&do=view&target=_README_GEOS-Chem.pdf)	✓	✓		✓	✓		
CU-Boulder GEOS-Chem adjoint v35f, 2.5° × 2° × 47 (Henze et al., 2007)	✓	✓		✓	✓	✓	✓
RAQMS, 1° × 1° × 35, free running (Pierce et al., 2007, 2009)	✓	✓	✓				
RAQMS, 1° × 1° × 35, with satellite assimilation (Pierce et al., 2007, 2009)	✓						
OsloCTM3 v2, ~2.8° × 2.8° × 60 (Søvde et al., 2012)	✓	✓		✓	✓	✓	✓
ECMWF C-IFS, ~0.7° × 0.7° × 54/1.125° × 1.125° × 54 (Flemming et al., 2015)	✓	✓		✓	✓	✓	✓

(b) Boundary condition model, resolution: lon× lat× vertical layer	BASE	EASALL (−20 %)	EASALL (−100 %)
SNU–GEOS-Chem v9-01-03, 2.5° × 2° × 47	✓	✓	
RAQMS, 1° × 1° × 35, free running	✓	✓	✓
RAQMS, 1° × 1° × 35, with satellite assimilation	✓		
ECMWF C-IFS, 1.125° × 1.125° × 54	✓	✓	

(c) Model	Meteorology	Biogenic VOCs; NO_*x*_	Lightning	Biomass burning	Chemical mechanism
GEOS-Chem	GEOS-5	MEGAN v2.1 (Guenther et al., 2012; Wang et al., 2009)	based on GEOS-5 deep convective cloud top heights and climatological observations (Murray et al., 2012)	GFED v3.0 (van derWerf et al., 2010)	GEOS-Chem standard NO_*x*_-O_*x*_ -hydrocarbon-aerosol (http://acmg.seas.harvard.edu/geos/doc/archive/man.v9-01-03/appendix_1.html)
RAQMS	Online (Pierce et al., 2007)				CB-IV (Gery et al., 1989) with adjustments
ECMWF C-IFS	IFS	MEGAN–MACC (Sindelarova et al., 2014); POET database for 2000 (Granier et al., 2005)	based on IFS convective precipitation (Meijer et al., 2001)	GFAS v1.0 (Kaiser et al., 2012)	CB05 (Yarwood et al., 2005)
STEM	WRF-ARW v3.3.1	WRF-MEGAN v2.1	based on scaled WRF convective precipitation	FINN v1.0 (Wiedinmyer et al., 2011)	SAPRC99 (Carter, 2000)

Acronyms: CAM-Chem: Community Atmosphere Model with Chemistry. C-IFS: Composition-Integrated Forecasting System. ECMWF: European Centre for Medium-Range Weather Forecasts. EMEP: European Monitoring and Evaluation Programme. GEOS-Chem: Goddard Earth Observing System with Chemistry. RAQMS: Realtime Air Quality Modeling System. SNU: Seoul National University. CB: Carbon Bond. FINN: Fire INventory from NCAR. GFAS: Global Fire Assimilation System. GFED: Global Fire Emissions Database. IFS: Integrated Forecasting System. MACC: Monitoring Atmospheric Composition and Climate. MEGAN: Model of Emissions of Gases and Aerosols from Nature. POET: Precursors of Ozone and their Effects in the Troposphere. WRF-ARW: Advanced Research Weather Research and Forecasting Model.

**Table 2:** (**a**) Evaluation of the period-mean (1 May–30 June 2010) multi-global model free simulations against the CASTNET observations, only at the sites where 95% of the hourly O_3_ observations are available. Evaluation of the individual models is summarized in (**b**). (**b**) Evaluation of the period-mean (May–June 2010) global model free simulations against the EANET and CASTNET observations. The STEM boundary condition models are highlighted in bold. (**c**) Evaluation of the period-mean (May–June 2010) multi-global model free simulations against the EANET observations in Japan and South Korea. Evaluation of the individual models is summarized in (**b**).

(a) Subregion	US EPA regions contained	Number of sites	Mean bias (ppbv)		RMSE (ppbv)	

			3 BC* models	8 global models	3 BC models	8 global models
Western US	8, 9, 10	19	−5.68	−2.52	10.37	7.05
Southern US	4, 6	18	11.61	10.24	13.62	11.96
Midwest	5, 7	13	8.03	7.66	9.16	8.67
Northeast	1, 2, 3	17	9.55	10.63	10.28	11.24
All	1–10	67	5.49	6.22	11.11	9.96

(b) Network	Number of sites	RMSE (ppbv)							

		CAM- Chem	EMEP	CHASER	SNU–GEOS- Chem	GEOS-Chem adjoint	RAQMS	OsloCTM3 v2	C-IFS
CASTNET	67	13.30	11.61	15.43	**15.55**	13.48	**9.32**	11.05	**11.00**
EANET	11	10.38	9.96	11.39	**9.18**	11.04	**8.60**	12.97	**10.86**

(c) Country	Number of sites	Mean bias (ppbv)		RMSE (ppbv)	

		3 BC* models	8 global models	3 BC models	8 global models
Japan	8	0.36	1.01	8.77	9.25
South Korea	3	1.14	3.98	8.37	10.51
All	11	0.57	1.82	8.66	9.61

* BC: boundary conditions.

**Table 3:** (**a**) Evaluation of the hourly STEM-simulated total O_3_ (averaged from the three base simulations that used the different free-running boundary conditions) against the CASTNET surface observations for 8 May–30 June 2010. The subregional-mean *R*(O_3_, EAS, 20 %) and its correlation coefficient with the observed O_3_ are also shown. **(**b**)** Evaluation of the hourly STEM-simulated total O_3_ (separately for three base simulations that used the different free-running boundary conditions) against the CASTNET surface observations for 8 May–30 June 2010.

(a) Subregion	US EPA regions contained	Number of sites	Mean elevation (km): actual/ model	Mean bias (ppbv)	RMSE (ppbv)	Correlation (model base; obs)	Correlation (obs; modeled EAS)	Mean EAS sensitivity (ppbv)
Western US	8, 9, 10	22	1.75/1.71	1.60	4.86	0.76	0.34	0.48
Southern US	4, 6	22	0.38/0.31	20.33	22.13	0.58	0.27	0.15
Midwest	5, 7	16	0.29/0.28	15.64	17.97	0.70	0.15	0.17
Northeast	1, 2, 3	20	0.36/0.26	20.94	24.16	0.47	0.17	0.21
All	1–10	80	0.73/0.68	16.17	18.30	0.66	0.13	0.20

(b) Subregion	US EPA regions contained	Number of sites	Mean bias (ppbv)/RMSE (ppbv)/ correlation (model base; obs)		

			SNU–GEOS-Chem	C-IFS	RAQMS
Western US	8, 9, 10	22	1.68/4.83/0.77	4.16/6.63/0.70	−1.03/4.81/0.76
Southern US	4, 6	22	21.18/22.94/0.57	20.34/22.07/0.60	19.48/21.45/0.56
Midwest	5, 7	16	15.77/18.17/0.70	16.41/18.46/0.72	14.73/17.35/0.69
Northeast	1, 2, 3	20	21.25/24.36/0.47	21.86/24.80/0.48	19.71/23.40/0.45
All	1–10	80	16.57/18.62/0.66	16.89/18.84/0.67	15.03/17.52/0.64

**Table 4:** The ranges and standard deviations (ppbv, separated by “;”) of *R*(O_3_, [source region], 20 %) by 6–8 global models (defined in Eqs. 1a–1d), summarized by months in 2010. The monthly multi-model mean values are shown in Figs. 6–7.

Month/source region	All Foreign/ non-NAM (ppbv)	EUR (ppbv)	EAS (ppbv)	SAS (ppbv)
January	0.38–1.69; 0.41	0.002–0.12; 0.05	0.02–0.72; 0.24	0.001–0.11; 0.04
February	0.92–2.07; 0.37	0.02–0.15; 0.05	0.16–0.91; 0.28	0.02–0.12; 0.04
March	1.30–2.37; 0.38	0.07–0.21; 0.06	0.24–1.03; 0.30	0.03–0.12; 0.03
April	1.42–2.46; 0.33	0.09–0.23; 0.05	0.33–1.07; 0.28	0.04–0.12; 0.03
May	1.24–1.91; 0.21	0.06–0.17; 0.04	0.24–0.75; 0.19	0.05–0.11; 0.02
June	1.03–1.41; 0.13	0.03–0.07; 0.02	0.14–0.39; 0.09	0.04–0.07; 0.01
July	0.86–1.18; 0.13	0.02–0.04; 0.01	0.08–0.22; 0.06	0.01–0.04; 0.01
August	0.80–1.19; 0.13	0.01–0.04; 0.01	0.07–0.20; 0.05	0.02–0.04; 0.01
September	0.85–1.18; 0.13	0.03–0.05; 0.01	0.10–0.25; 0.06	0.02–0.06; 0.01
October	0.96–1.31; 0.14	0.04–0.10; 0.02	0.17–0.42; 0.09	0.03–0.08; 0.02
November	0.90–1.48; 0.19	0.05–0.15; 0.04	0.17–0.54; 0.14	0.04–0.10; 0.02
December	0.73–1.67; 0.29	0.03–0.18; 0.05	0.14–0.66; 0.19	0.04–0.12; 0.03
