# Supplementary material for: Impact of intercontinental pollution transport on North American ozone air pollution: an HTAP phase 2 multi-model study
Source: Atmos Chem Phys. Author manuscript; Available in PMC 2018 May 16. (PMC5954439; doi:10.5194/acp-17-5721-2017)
Supplement: Sup 1 [file NIHMS940844-supplement-Sup_1.pdf]

Supplement of Atmos. Chem. Phys., 17, 5721–5750, 2017
http://www.atmos-chem-phys.net/17/5721/2017/
doi:10.5194/acp-17-5721-2017-supplement
© Author(s) 2017. CC Attribution 3.0 License.

Atmospheric Chemistry and Physics
Open Access

Supplement of

Impact of intercontinental pollution transport on North American ozone air pollution: an HTAP phase 2 multi-model study

Min Huang et al.

Correspondence to: Min Huang (mhuang10@gmu.edu)

The copyright of individual parts of the supplement might differ from the CC-BY 3.0 licence.

GEOS-Chem:

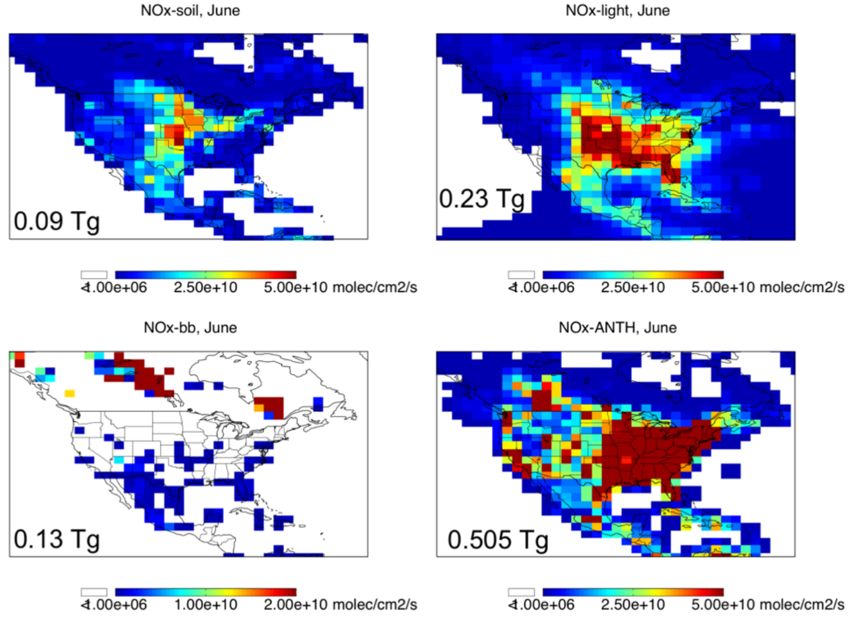

STEM:

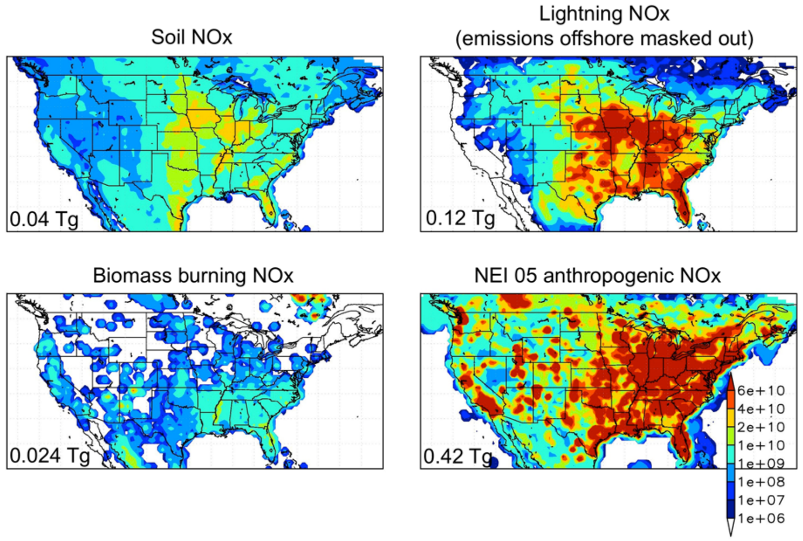

Figure S1. June 2010 soil, lightning, biomass burning and NEI05 anthropogenic NO_x emissions (in molecule/cm^2/s) used by GEOS-Chem and STEM in the Lapina et al. (2014) study, and the numbers at the lower-left corners indicate the domain integrated total amounts (note that GEOS-Chem emissions were plotted/intergated over a slightly larger domain than STEM's). The same set of non-anthropogenic emissions was used in the HTAP2 simulations.

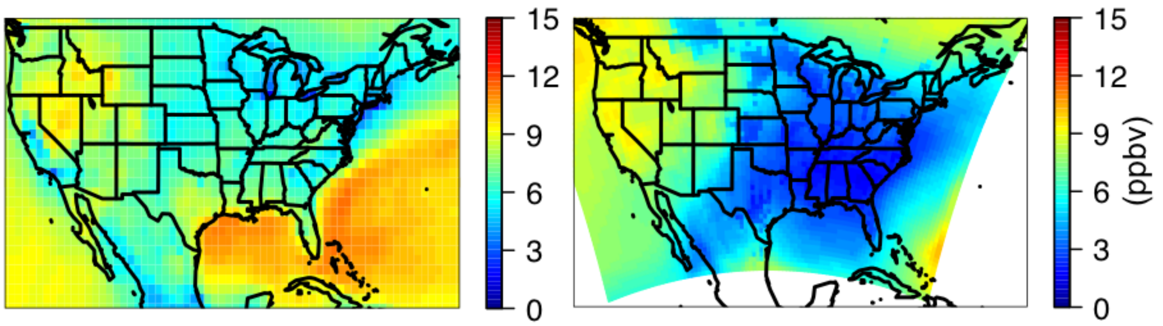

Figure S2. RAQMS mean surface O_3 changes (ppbv) due to assimilating OMI and MLS O_3 (left) and the resulting O_3 changes in STEM O_3 (right) in June 2010.

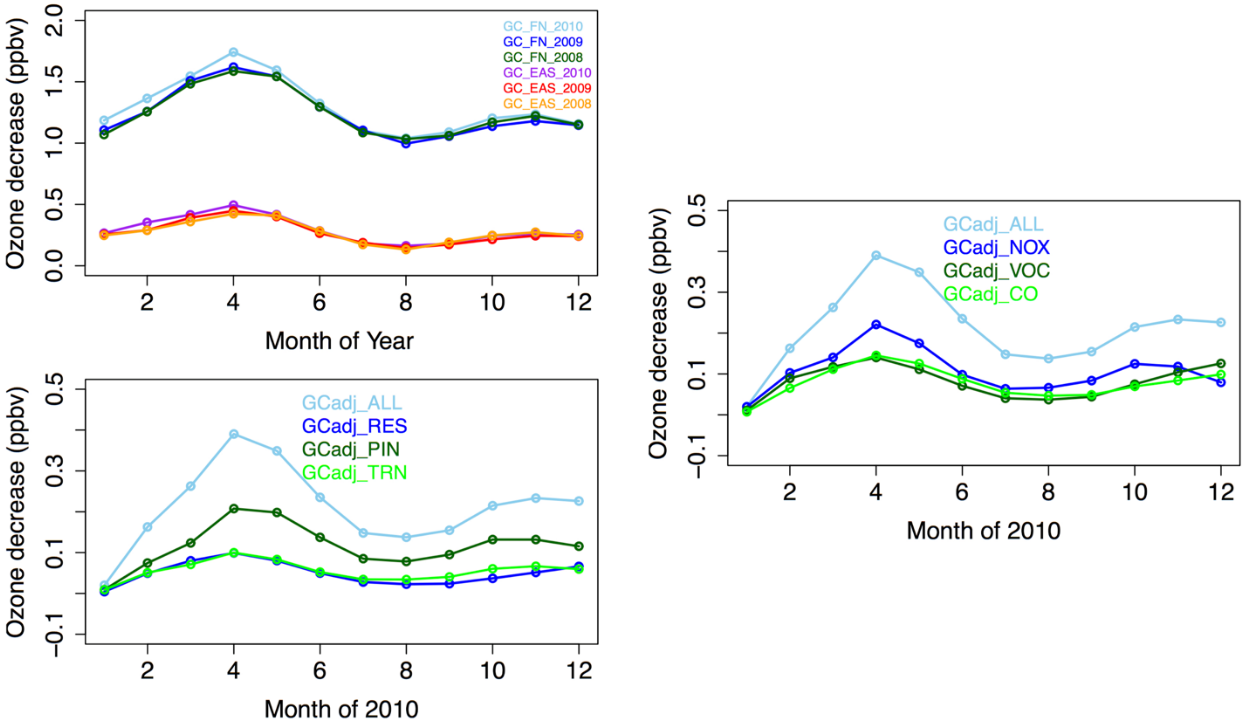

Figure S3. (Upperleft) North American surface R(O_3, EAS, 20%) and R(O_3, all-non NAM, 20%) from SNU GEOS-Chem simulation, summarized by year. GEOS-Chem adjoint estimated North American surface R(O_3, EAS, 20%), in response to emission perturbations of the individual emission sector (lowerleft, RES: residential; PIN: power and industry; TRN: transportation), and different O_3 precursors (right).

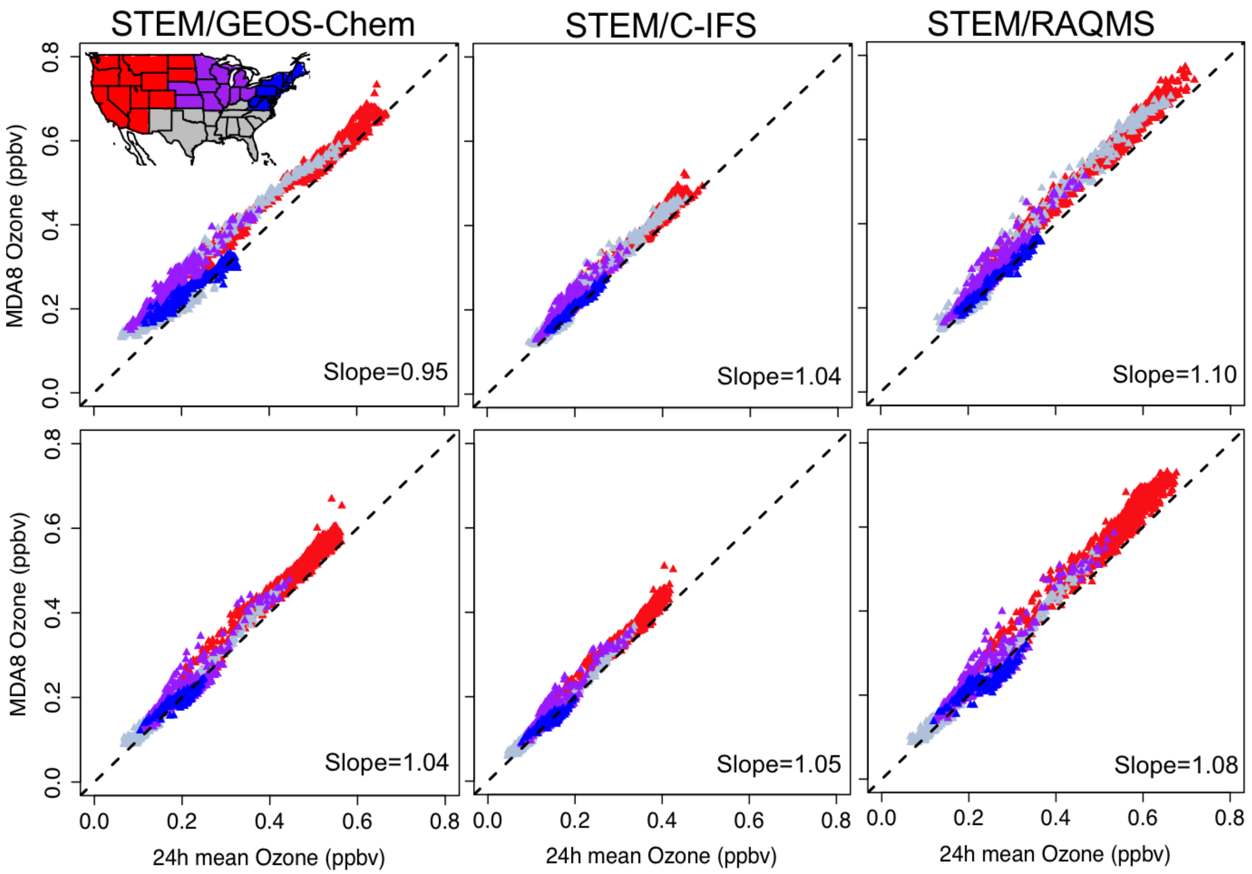

Figure S4. Scatterplots of STEM MDA8 and all-time R(O_3, EAS, 20%) for (upper) May and (lower) June 2010 in all US model grids, colored by four different US subregions. The subregions are defined in the inset of the upperleft panel, also consistent with the definitions in Figure 2/Tables 2-3. The results from the STEM runs using (left) GEOS-Chem, (middle) ECMWF C-IFS and (right) RAQMS boundary conditions are shown separately.

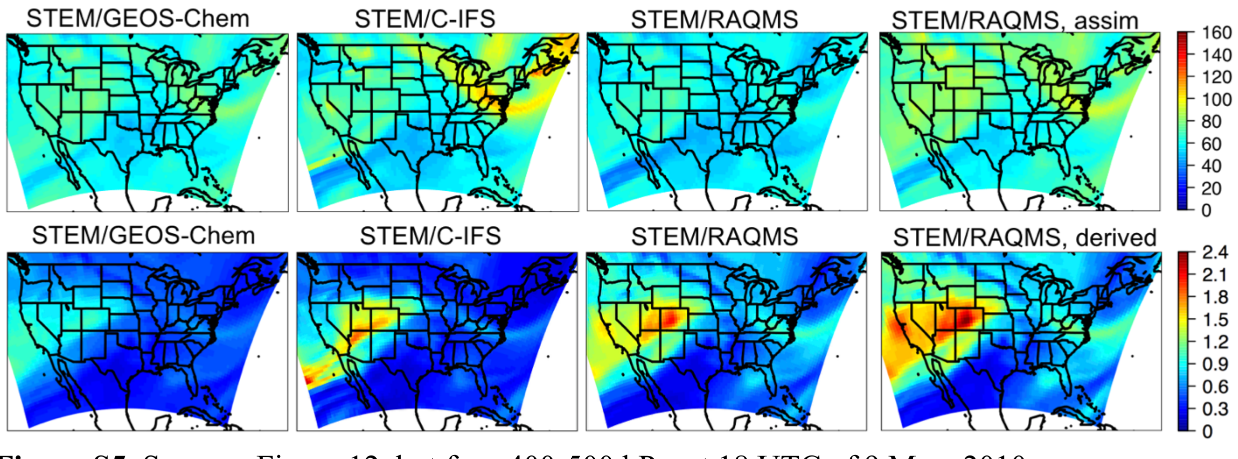

Figure S5. Same as Figure 12, but for ~400-500 hPa, at 18 UTC of 9 May, 2010.

Table S1. HTAP2 regional and global anthropogenic emissions from various sectors for NO_x, NMVOCs, and CO, in tons/year. Source: http://edgar.jrc.ec.europa.eu/htap_v2/index.php

Year	Species	Region	Energy (Power)	Industry	Residential	Transportation
2008	NO_x	World	2.67E+07	2.19E+07	6.38E+06	4.23E+07
		MICS Asia	1.25E+07	1.20E+07	3.19E+06	1.74E+07
		US+Canada	3.41E+06	4.68E+06	9.59E+05	8.42E+06
2010		World	2.77E+07	2.30E+07	6.59E+06	4.18E+07
		MICS Asia	1.34E+07	1.37E+07	3.30E+06	1.80E+07
		US+Canada	3.09E+06	4.17E+06	9.81E+05	7.38E+06
2008	NMVOCs	World	1.16E+06	6.61E+07	4.19E+07	3.46E+07
		MICS Asia	4.81E+05	2.05E+07	2.17E+07	1.62E+07
		US+Canada	5.95E+04	9.47E+06	1.63E+06	5.63E+06
2010		World	1.17E+06	6.68E+07	4.30E+07	3.45E+07
		MICS Asia	4.99E+05	2.31E+07	2.22E+07	1.73E+07
		US+Canada	5.40E+04	8.97E+06	1.57E+06	4.61E+06
2008	CO	World	8.44E+06	1.39E+08	2.33E+08	1.70E+08
		MICS Asia	5.04E+06	9.95E+07	1.47E+08	6.16E+07
		US+Canada	7.51E+05	6.70E+06	1.55E+07	4.27E+07
2010		World	8.91E+06	1.37E+08	2.39E+08	1.60E+08
		MICS Asia	5.44E+06	9.96E+07	1.51E+08	6.03E+07
		US+Canada	6.82E+05	5.79E+06	1.47E+07	3.42E+07
